# Clinical Characteristics and Incidence of Hemorrhagic Complications in Patients Taking Factor Xa Inhibitors in Spain: A Long-Term Observational Study

**DOI:** 10.3390/jcm13061677

**Published:** 2024-03-14

**Authors:** Carlos Escobar, Beatriz Palacios, Miriam Villarreal, Martín Gutiérrez, Margarita Capel, Ignacio Hernández, María García, Laura Lledó, Juan F. Arenillas

**Affiliations:** 1 Cardiology Department, University Hospital La Paz, 28046 Madrid, Spain; 2 AstraZeneca Farmacéutica, 28050 Madrid, Spain; beatriz.palacios@astrazeneca.com (B.P.); miriam.villarreal@astrazeneca.com (M.V.); martin.gutierrez@astrazeneca.com (M.G.); margarita.capel@astrazeneca.com (M.C.); 3 Atrys Health, 28002 Madrid, Spain; ihernandez@atryshealth.com (I.H.); mgmarquez@atryshealth.com (M.G.); llledo@atryshealth.com (L.L.); 4 Neurology Department, Comprehensive Stroke Center, Hospital Clínico Universitario, 47003 Valladolid, Spain; juanfarenillas@gmail.com; 5 Clinical Neurosciences Research Group, Department of Medicine, University of Valladolid, 47003 Valladolid, Spain

**Keywords:** apixaban, bleeding, edoxaban, enoxaparin, Factor Xa inhibitors, rivaroxaban

## Abstract

**Objective.** To analyze the clinical characteristics of patients taking Factor Xa inhibitors (FXai), either direct FXai or enoxaparin (only in active cancer patients), and to estimate the incidence of and risk factors for major bleeding during FXai use. **Methods.** A retrospective cohort study, which included secondary data from computerized health records of primary care centers and hospitals in seven Spanish Autonomous Communities. **Results.** 9374 patients were analyzed, with 8972 taking direct FXai and 402 enoxaparin. At baseline, the mean age (SD) was 71.8 (9.4) years, 56.0% were women, 76.3% had hypertension, 33.6% had type 2 diabetes, and 25.5% had heart failure. The most common indication for FXai use was atrial fibrillation (72.3%), followed by venous thromboembolism (22.2%) and non-mechanical cardiac–valve replacement (5.6%). At the end of the follow-up period, the incidence rates of major bleeding overall, gastrointestinal, and intracranial were 10.2, 9.0, and 0.8 per 100 person-years, respectively. The total incidence of fatal major bleeding was 0.5 per 100 person-years. Incidence rates of all bleedings progressively decreased over time, with 62.5% of the first events occurring in the initial three months and reaching 76.8% within six months following initiation of treatment. Only 4.8% of the 1st major bleedings led to death, 2.3% in the case of major gastrointestinal bleeding, and 30.8% after an intracranial bleeding. 65.9% of patients discontinued anticoagulation after experiencing major bleeding. **Conclusions.** In Spain, patients taking FXai were old and had many comorbidities. Despite incidence rates of major bleeding were high, incidence rates of intracranial and fatal bleedings were low, but more efforts are required due to their relevant clinical impact.

## 1. Introduction

Anticoagulation is the cornerstone in the prevention of thromboembolic complications in patients with atrial fibrillation (AF), the prevention and treatment of acute venous thromboembolism (VTE), and the treatment of selected patients early after bioprosthetic valve implantation [1,2,3,4].

For these purposes, vitamin K antagonists (VKAs) have been traditionally used in AF patients and low-molecular-weight heparins, followed by VKAs among patients with VTE [1,2,3]. However, VKAs have many disadvantages (i.e., the requirement for frequent anticoagulation control, a narrow therapeutic window, certain dietary restrictions, drug–drug interactions, and particularly, a relatively high risk of bleeding) that have limited their use in clinical practice [5].

Fortunately, direct oral anticoagulants (DOACs) overcome the majority of these limitations. But, despite advancements in treatment and the lower risk of major bleeding reported in DOACs compared to VKAs [6,7], bleeding events remain the most concerning complication associated with anticoagulant therapy, and some major bleeding still leads to significant morbidity and mortality [8,9]. On the other hand, although the information provided by clinical trials is very valuable and DOACs are more manageable than VKA, it is important to ascertain the bleeding risk in real-world clinical practice [10].

On the other hand, patients with cancer have a high risk of VTE. Different head-to-head clinical studies have compared the use of DOACs vs. low-molecular-weight heparins in this population for the treatment and prophylaxis of VTE, and also in the AF population. In general, these studies have shown that DOACs could be a superior therapeutic option for managing cancer-associated thromboembolism compared to low-molecular-weight heparin. Despite these results, low-molecular-weight heparins, particularly enoxaparin, an anti-factor Xa, are still frequently used in Spain among patients with cancer [11,12,13,14].

The management of patients with major bleeding is complex, as many factors need to be considered, including the level of awareness of bleeding risks and the management of DOAC-related bleeds, including the use of specific reversal agents when required [15,16]. As a result, it is necessary to foster a more comprehensive approach to address anticoagulant-related bleeding complications. This will help patients and clinicians optimize the risks/benefits of anticoagulant use by identifying patients at high risk of major bleeding complications and allowing appropriate actions to be taken, such as treating modifiable risk factors for bleeding and reducing the intensity or stopping anticoagulant use if necessary [1,2,3,17].

The aim of this study was to analyze the clinical characteristics of patients taking Factor Xa inhibitors (FXai), either oral direct FXai (i.e., apixaban, edoxaban, or rivaroxaban) or enoxaparin (only in active cancer patients) both overall and as individual groups, as well as to estimate the incidence of and risk factors associated with major bleeding during FXai use. In addition, the incidence of fatal, intracranial, gastrointestinal, traumatic, and other major bleedings was also calculated.

## 2. Methods

A retrospective and observational cohort study, which included secondary data from computerized health records of primary care centers and hospitals in seven Spanish Autonomous Communities since 2012, was conducted using the BIG-PAC^®^ database. This is an administrative database, representative of the Spanish population [18,19,20]. In this study, all adult patients with a prescription of either an oral direct FXai (i.e., apixaban, edoxaban, or rivaroxaban) or enoxaparin (only in active cancer patients) in therapeutic dose, and a previous recording (or on the same day) of any of the following diagnoses: VTE, AF, or non-mechanical cardiac–valve replacement, during the study period (i.e., between 1 January 2013 and 31 December 2022) were included. In addition, to be enrolled, patients had to be at least 18 years of age and have at least 12 months of data availability in the database before the prescription of the anticoagulant therapy. In contrast, patients were excluded if any of the following criteria applied: oral direct FXai or enoxaparin use within 60 days before the start of chronic use of these products for the study indications within the study period, history of oral direct FXai or enoxaparin use before the first recording of a diagnosis under study, a major bleeding event within 60 days before the direct FXai or enoxaparin initiation, palliative care initiation any time before the FXai initiation, or pregnancy at the time of direct FXai or enoxaparin initiation. Since this was an observational study, no specific diagnostic or therapeutic procedures were performed for inclusion in the study. The research was performed in accordance with the Declaration of Helsinki. The study was approved by the Research Ethics Committee of Consorci Sanitari de Terrassa, Barcelona, Spain. The requirement for obtaining written informed consent was waived by the Research Ethics Committee, since the study collected secondary data that were fully and completely anonymized.

The day of the first recording of oral FXai (apixaban, edoxaban, or rivaroxaban) or enoxaparin (only in active cancer patients) constituted the start of the observational period for each patient and the index date at which baseline characteristics were obtained. The end of the observational period was determined if any of the following situations occurred: discontinuation of oral FXai therapy or enoxaparin (only in active cancer patients), switching to or concomitant use of a non FXai anticoagulant, recording of pregnancy, completion of the study period (i.e., 31 December 2022), disenrollment from the database, end of data collection, patient death, or initiation of palliative care.

At baseline, data from biodemographics (age, sex, body mass index, alcohol use, CHA2DS2-VASc score, HAS-BLED score, and updated Charlson comorbidity index), cardiovascular risk factors (hypertension, hypercholesterolemia, diabetes, smoking), vascular disease (heart failure, chronic kidney disease, coronary artery disease, peripheral artery disease, and cerebrovascular disease), and other comorbidities (chronic pulmonary disease, cancer, anemia, major bleeding history >60 days prior to FXai start, liver disease, peptic ulcer disease) were collected. Clinical conditions were defined using medical codes entered by practices, using the International Classification of Diseases (ICD), Ninth Revision (ICD-9), and Tenth Revision (ICD-10) (all converted to ICD-10 in the database) (Supplementary Table S1). In addition, FXai indications (VTE, AF, and non-mechanical cardiac–valve replacement), the date of FXai initiation, compliance with treatment during the follow-up period, discontinuation after major bleeding, and DOAC dose modifications according to the summary of product characteristics (Supplementary Table S2) [21,22,23,24], were also recorded. Laboratory tests closest to the index date were collected and included hemoglobin, HbA1c, platelet count, and renal function (estimated glomerular filtration rate and creatinine clearance). Concomitant treatments (within 120 days prior to enrolment), including gastroprotective drugs, antihypertensive therapies, lipid lowering, antidiabetic, non-steroidal anti-inflammatory, and antiplatelet and anticancer drugs (within 180 days prior to enrolment), were also captured. Although this may be considered a limitation, as it does not only assess concomitant treatments at the moment of enrolment, this time window was chosen to assess their impact on the incidence of bleeding. Medication use was derived from issued prescriptions coded using anatomical therapeutic chemical codes (ATC medication code: “A10”). Data were analyzed in the overall study population according to the type of FXai, the dose of DOAC (as per label, higher or lower than label), the type of DOAC, history of major bleeding during the follow-up period, and the indication at the first prescription.

The first outcome was the first major bleeding. Major bleeding was defined as fatal bleeding or bleeding that causes hospitalization. Major bleeds were categorized as gastrointestinal, intracranial, or other life-threatening bleeding. Major bleeding incidence rates were calculated as the total number of incident major bleeding events divided by the total person-time at risk, calculated in the overall study period and cumulatively at 3, 6, 12, 24, and 36 months. Incidence rates of major bleeding, including those occurring in critical sites (defined in Supplementary Table S1), and those that were fatal were calculated for the overall study population and according to the type of FXai, the dose of DOAC, and the indication at first prescription. Additionally, incidence rates of major gastrointestinal bleeding and intracranial bleeding (including fatal cases) were also calculated for the overall study population and according to the type of FXai. Following a similar approach, event rates were also calculated by dividing the total number of bleeding events, including recurrent events, by the total person-time of follow-up. Finally, risk factors for major bleeds were calculated for patients taking DOACs.

## 3. Statistical Analysis

Demographic and clinical data were summarized using descriptive statistics. Qualitative data were presented by their absolute and relative frequencies. Quantitative data were presented using the mean and standard deviation. In the bivariate analysis, Student’s *t*-test was used to compare the two means, the Chi-square test was used to compare categorical variables, and the Z test was used for 2-proportion comparisons (comorbidities). Incidence rates for bleeding events were reported per 100 person-years with 95% confidence intervals. Survival curves (Kaplan–Meier) were calculated for major bleeding and fatal bleeding in the overall population, DOACs, and enoxaparin groups, as well as for DOACs according to label prescription. In addition, survival curves (Kaplan–Meier) were also calculated for major and fatal gastrointestinal bleedings and for major and fatal intracranial bleedings in the overall population, DOACs, and enoxaparin groups. The log-rank test was used to estimater whether there were significant differences between groups. On the other hand, the strength of association between potential risk factors and major bleeds for DOAC patients was calculated using COX regression models including all potential risk factors, estimating the Odds Ratio along with their 95% confidence intervals. The results were presented in a forest plot figure. Statistical significance was established at *p* < 0.05. All statistical procedures were performed using Stata MP Version 14.2 (StataCorp LLC., College Station, TX, USA).

## 4. Results

**Figure 1:**
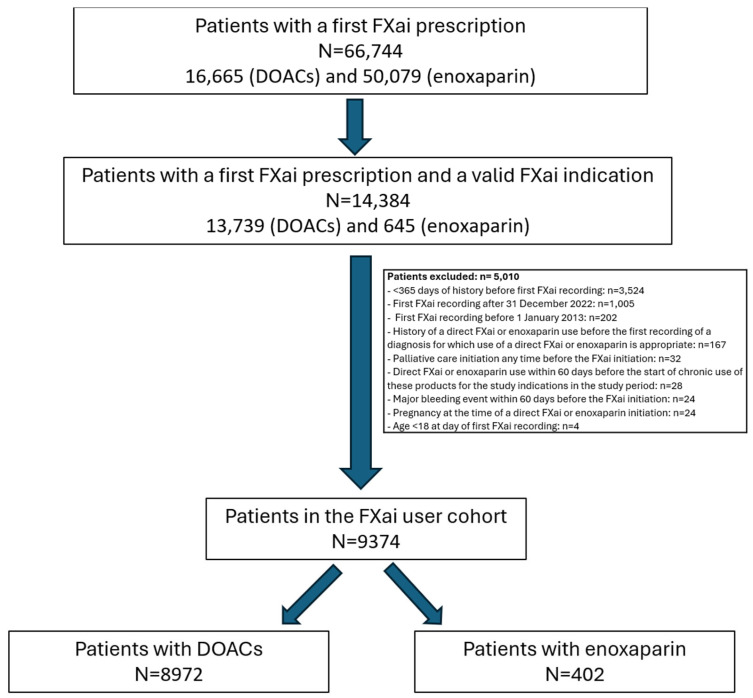
Flow chart of the study. DOACs: direct oral anticoagulants; FXai: factor Xa inhibitors.

Out of 1.9 million patients of the BIG-PAC^®^ database, a total of 66,744 patients received their first FXai prescription (16,665 taking DOACs and 50,079 taking enoxaparin), of whom 14,384 had a study indication (13,739 and 645, respectively). After excluding 5010 patients due to different reasons, 9374 patients were finally analyzed, with 8972 taking DOACs and 402 taking enoxaparin (only in active cancer patients) (Figure 1).

Baseline clinical characteristics of the overall study population are shown in Table 1. The mean age (SD) was 71.8 (9.4) years, 56.0% of patients were women, 76.3% had hypertension, 33.6% had type 2 diabetes, 25.5% had heart failure, 15.8% had chronic kidney disease, 15.5% had coronary artery disease, and 6.4% had prior cerebrovascular disease. The most common indication for the use of FXai was AF (72.3%), followed by VTE (22.2%) and non-mechanical cardiac–valve replacement (5.6%). During the study period, the proportion of patients initiating FXai use increased from 2.8% in 2013 to 23.0% in 2022. Of patients treated with DOAC, 15.6% received doses higher or lower than the label, compliance ≥80% was achieved in 91.7% of patients, and 65.9% of patients discontinued anticoagulation after experiencing major bleeding. As the majority of patients in our study were taking DOACs, the clinical profile was very similar to that of the overall population. The greater increase in the prescription of DOAC was seen mainly in the last three years, mainly during the COVID-19 pandemic, as no anticoagulation control was required (compared to VKA). Baseline clinical characteristics were also analyzed according to the development of major bleeding during the study period. Among patients taking DOACs, there was a trend towards a higher age in those who experienced a major bleeding event (77.4 (9.62) vs. 71.5 (9.3); *p* = 0.07), which was seen as a shift towards greater percentages in higher age groups (<0.001). In the case of patients receiving enoxaparin only, age differences (75.9 (11.0) vs. 72.0 (9.4)) reached statistical significance (*p* = 0.002). No other relevant differences were observed. Additionally, baseline data according to the type of FXai, the type of DOAC, the dose of DOAC (per label, higher or lower than the label), the indication at first prescription, and CHA2DS2-VASc and HAS-BLED scores were also provided in Table 1 and Supplementary Tables S3 and S4. No relevant differences were found between groups.

**Figure 2:**
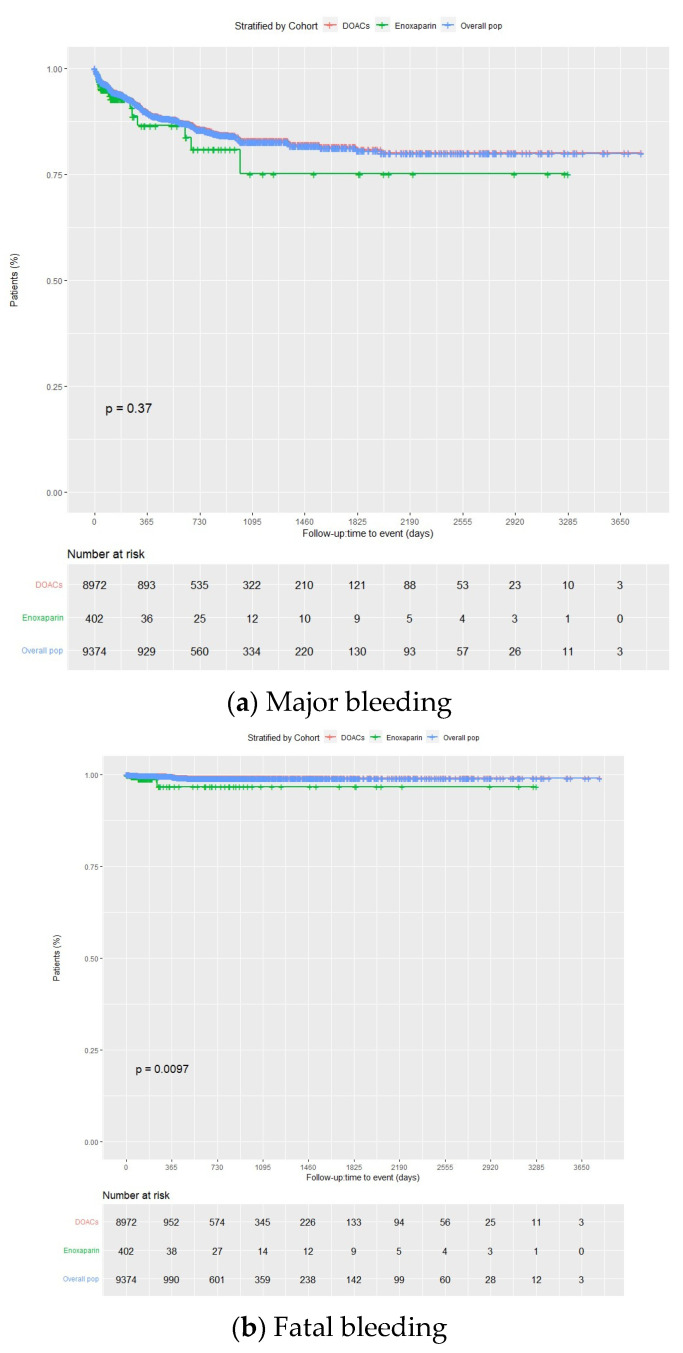
Kaplan–Meier curves for major and fatal bleedings for the overall population, DOACs, and enoxaparin groups. DOACs: direct oral anticoagulants.

**Figure 3:**
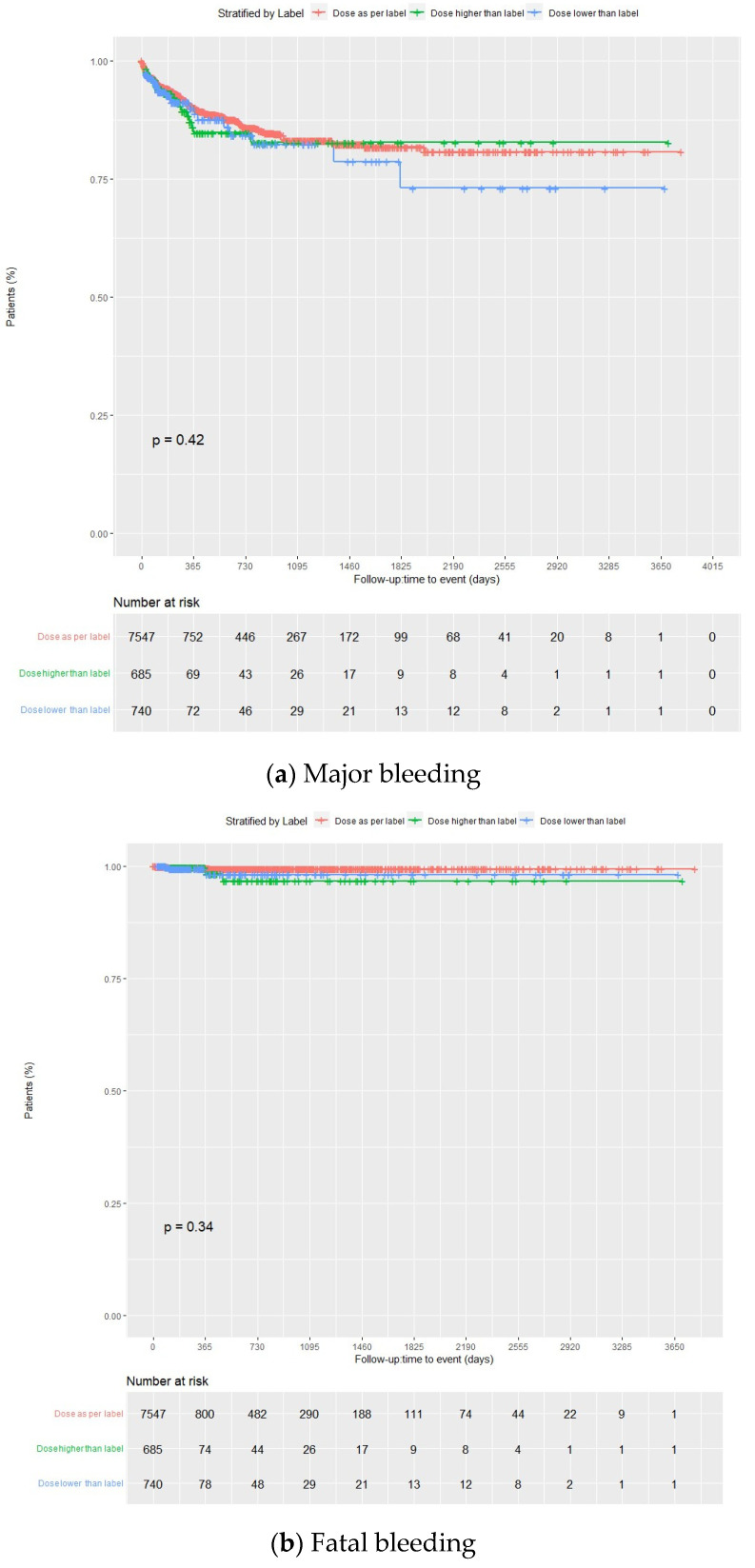
Kaplan–Meier curves for major and fatal bleedings for DOACs, according to label prescription. DOACs: direct oral anticoagulants.

Overall, 5.3% of the patients presented with major bleeding, with incidence rates at the end of the follow-up period of 10.2 (95% CI 9.6–10.8), 10.1 (95% CI 9.5–10.7), and 13.1 (95% CI 9.8–16.4) per 100 person-years in the overall group, DOACs, and enoxaparin, respectively. Among patients taking DOACs, 5.2% experienced major bleeding (88.9% from gastrointestinal bleeding, 6.8% from intracranial hemorrhage, and 4.3% from others), with incidence rates of 10.1 (9.5–10.7) per 100 person-years for the full group, and 9.9 (95% CI 9.2–10.6), 10.8 (95% CI 8.5–13.2), and 11.3 (95% CI 9.0–13.6) per 100 person-years when doses were prescribed as per label, higher than label, and lower than label, respectively. A total of 6.5% of enoxaparin patients suffered from major bleeding, with an incidence rate of 13.1 (95% CI 9.8–16.4). The incidence of major bleeding was similar between the enoxaparin and DOACs groups (*p* = 0.37) (Figure 2a and Figure 3a; Supplementary Table S5).

In all cases, the incidence rates of major bleeding progressively decreased over time, with 62.5%, 62.3%, and 65.3% experiencing the first major bleeding within the first three months of treatment (about 77% within the first six months) in the overall group, DOACs, and enoxaparin groups, respectively. There were only 72 rebleedings in the overall population, likely due to the high FXai discontinuation rate following the first bleeding (65.9%, 65.7% and 69.2% for the overall, DOACs, and enoxaparin groups). At the end of the follow-up period, the incidence rates of fatal bleeding were 0.5 (95% CI 0.4–0.6), 0.4 (95% CI 0.3–0.6), and 2.0 (95% CI 0.6–3.4) per 100 person-years for the total, DOACs, and enoxaparin groups, respectively (4.8%, 4.3%, and 15.4%, respectively, of the total major bleeding cases). Incidence rates for critical site bleeding were 0.8 (95% CI 0.7–1.0), 0.8 (95% CI 0.6–0.9), and 3.0 (95% CI 1.4–4.7) per 100 person-years, respectively. In all cases, the incidence rates of critical site bleeding and fatal bleeding progressively decreased over time. Among patients taking DOACs, incidence rates of fatal bleeding were 0.4 (95% CI 0.2–0.5), 0.6 (95% CI 0–1.1), and 0.8 (95% CI 0.1–1.4) per 100 person-years when the dose was prescribed as per label, higher than label, and lower than label, respectively. The incidence of fatal bleeding was higher with enoxaparin than with DOACs (*p*= 0.0097) (Figure 2b and Figure 3b; Supplementary Table S5). Major, critical site, and fatal bleeding incidence rates according to FXai indication and the type of direct FXai were also provided in Supplementary Table S5.

**Figure 4:**
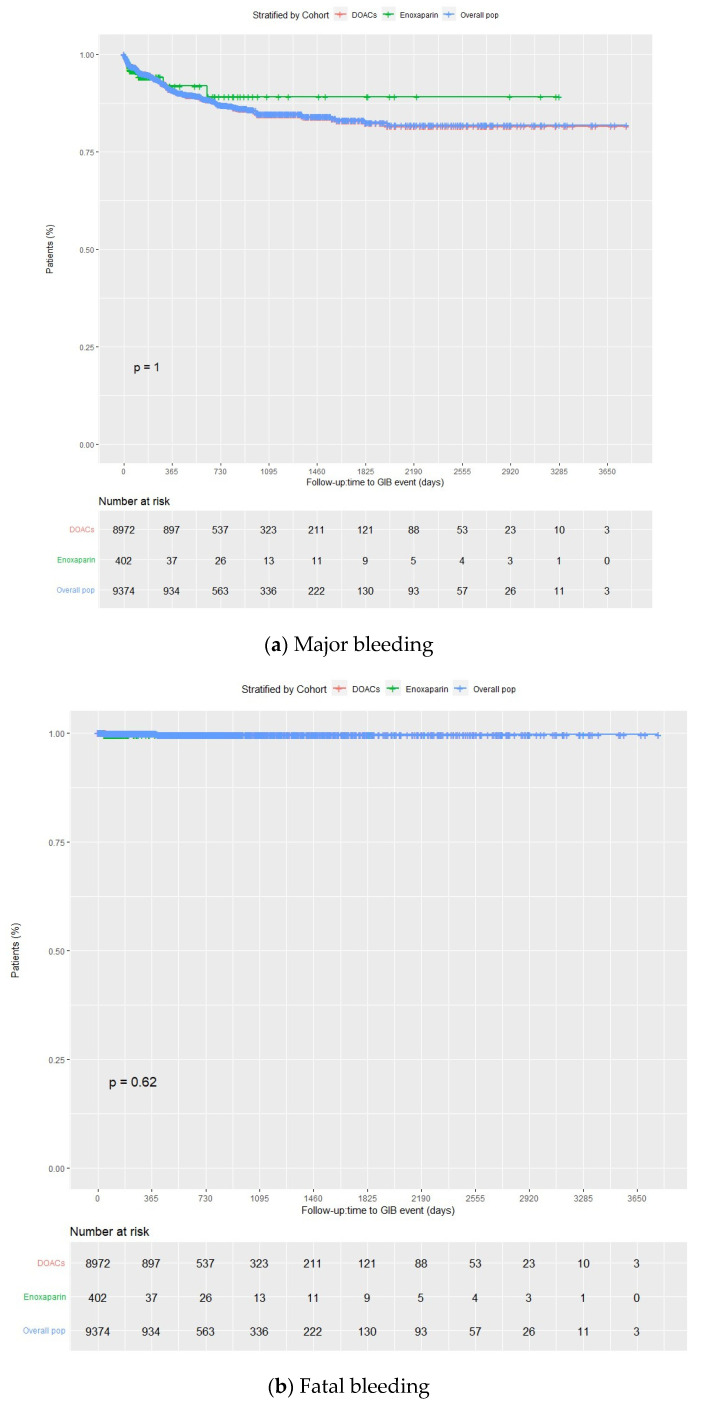
Kaplan–Meier curves for major and fatal gastrointestinal bleedings for the overall population, DOACs, and enoxaparin groups. DOACs: direct oral anticoagulants.

The overall incidence rates of major and fatal gastrointestinal bleeding at the end of the follow-up period were 9.0 (95% CI 8.4–9.5) (87.9% of the total major bleedings) and 0.2 (95% CI 0.1–0.3) per 100 person-years (2.3% of the total major gastrointestinal bleeding), respectively, decreasing over time (Figure 4a,b, Supplementary Table S6). Upper gastrointestinal bleeding was more common than lower gastrointestinal bleeding (Supplementary Table S6).

**Figure 5:**
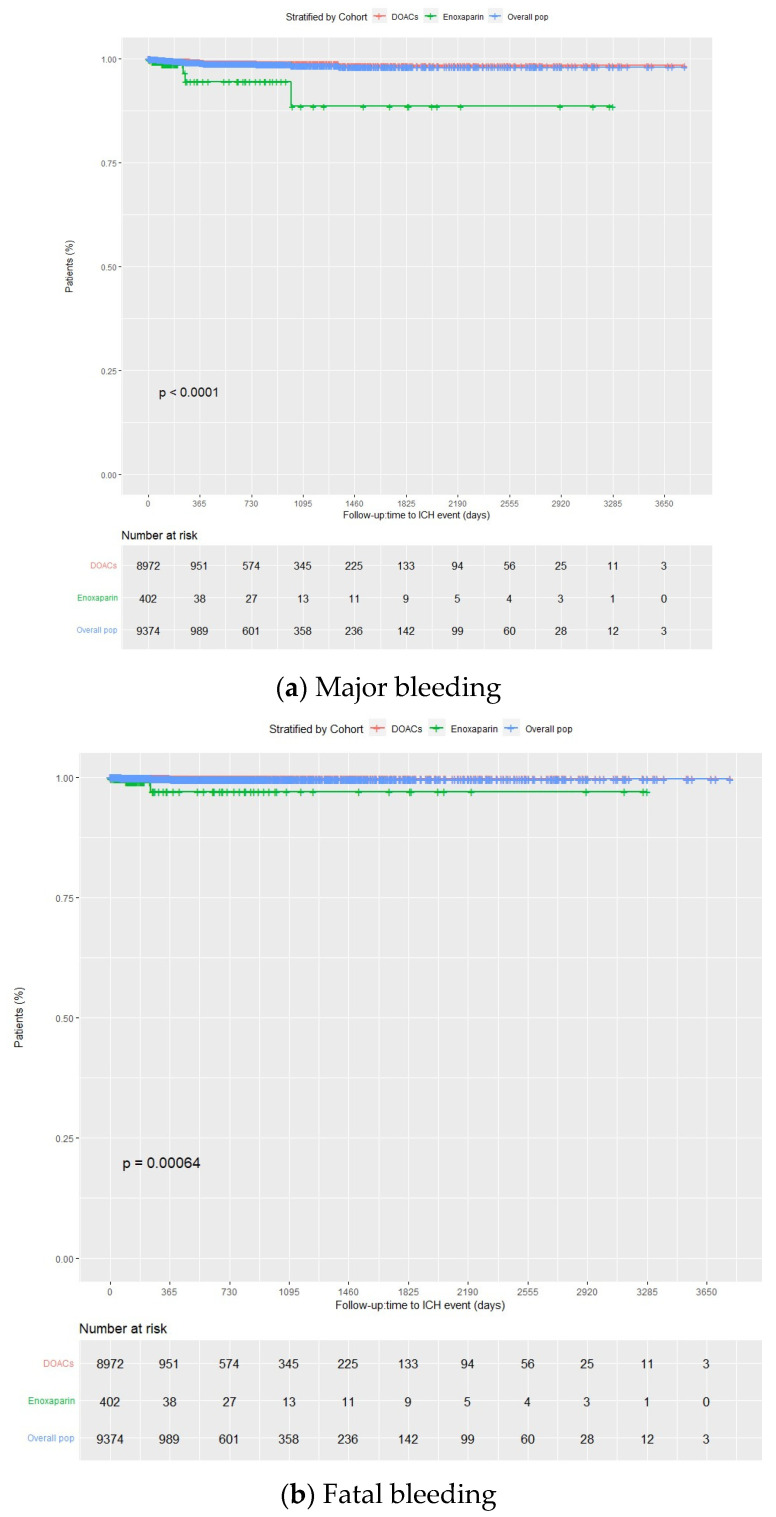
Kaplan–Meier curve for major and fatal intracranial bleedings for the overall population, DOACs, and enoxaparin groups. DOACs: direct oral anticoagulants.

The overall incidence rates of major and fatal intracranial bleeding were 0.8 (95% CI 0.6–1.0) (7.9% of the total major bleedings) and 0.3 (95% CI 0.2–0.4) per 100 person-years (30.7% of the total intracranial bleeding), respectively, decreasing over time (Figure 5a,b, Supplementary Table S7). Intracerebral bleeding was more common than subarachnoid hemorrhage (Supplementary Table S7).

In addition, at the end of the follow-up period, among patients taking DOACs, the incidence rates of intracranial and gastrointestinal bleeding were 0.7 (95% CI 0.5–0.9) and 9.0 (95% CI 8.4–9.6) per 100 person-years, respectively. In the case of patients taking enoxaparin, these rates were 3.5 (95% CI 1.7–5.3) and 9.1 (95% CI 8.5–9.7) per 100 person-years, respectively. Whereas no significant differences were observed between enoxaparin and DOACs regarding gastrointestinal bleeding, major and fatal intracranial bleedings were more common with enoxaparin than with DOACs (*p* < 0.001 and *p* = 0.00064, respectively) (Figure 4a,b and Figure 5a,b).

Incidence rates of trauma-related bleedings and other major bleedings are shown in Supplementary Tables S8 and S9.

**Figure 6:**
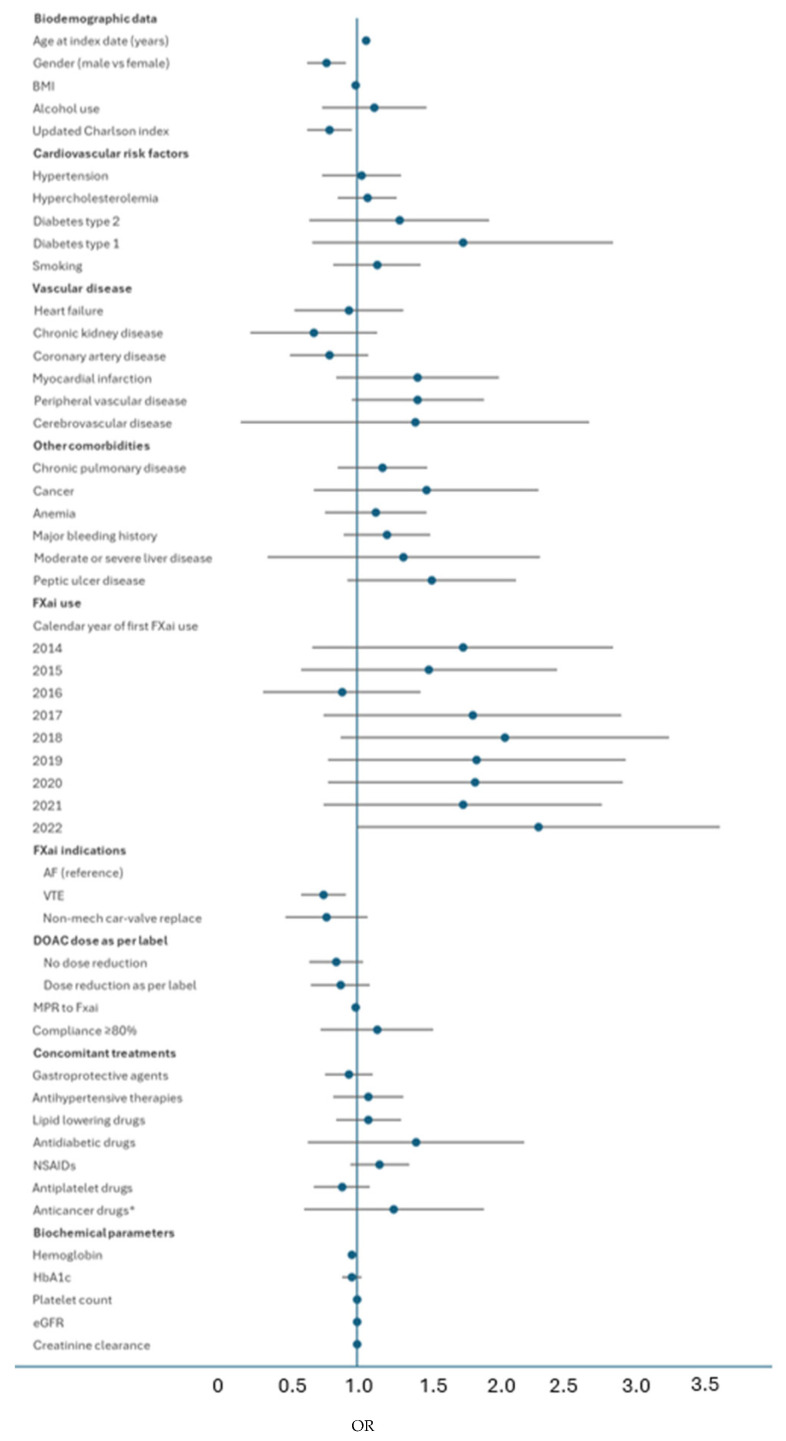
Risk factors for major bleeds for DOAC patients. * within 180 days prior to the index date. AF: atrial fibrillation; BMI: body mass index; DOACs: direct oral anticoagulants; eGFR: estimated glomerular filtration rate; FXai; Factor Xa inhibitors; MPR: medical possesion ratio; NSAIDs: non-steroidal anti-inflammatory drugs; OR: Odds Ratio; VTE: venous thromboembolism.

Risk factors for major bleeds among patients taking DOACs were specifically analyzed using a Cox Regression Model (Supplementary Table S10) and presented as a forest plot in Figure 6. Increasing age and discontinuation after major bleeding were independently associated with the risk of developing major bleeding. Of note, dose reduction of DOACs was not associated with a protective effect against the risk of major bleeding.

## 5. Discussion

Our study showed that patients initiating treatment with FXai, mainly for AF or VTE, were old and had many comorbidities. The overall incidence rates of major, gastrointestinal, intracranial, and fatal bleedings were 10.2, 9.0, 0.8, and 0.5 per 100 person-years, respectively. Of note, the incidence rates of all bleedings progressively decreased over time.

In our study, a total of 9374 patients with a valid FXai indication were included, of whom 8972 were taking DOACs, with the main indication being AF (72%), followed by VTE (22%). Disparities in the prescription of DOACs according to indication were related to the different epidemiologies of both conditions but also to the lack of reimbursement of DOACs in the case of VTE in Spain. In addition, the increase in the prescription of DOACs observed over time in our study was slow and lower than in other European countries, mainly due to restrictions on the prescription of DOACs in patients with AF, only limited to some particular conditions [25,26]. However, during the COVID-19 pandemic, the prescription of DOACs was accelerated (initial prescription and switching from VKA) to avoid visits for anticoagulation control, leading to a reduction in the risk of contagion [27].

With regard to the clinical characteristics of the study population, the mean age was 71.8 years, 56% were women, 76% had hypertension, one third had type 2 diabetes, one quarter had heart failure, and around 6% had prior cerebrovascular disease. The clinical profile of patients was similar regardless of the FXai indication, the type of Fxai, and the dose of DOACs. In phase 3 clinical trials comparing DOACs vs. warfarin among patients with AF (ROCKET-AF, ARISTOTLE, and ENGAGE AF-TIMI 48), the mean age was 70–73 years, 35–40% of patients were women, 90–94% had hypertension, 25–40% had diabetes, 35–63% had heart failure, and 19–52% had prior cerebrovascular disease [28,29,30]. However, the clinical profile of patients with VTE included in the phase 3 clinical trials comparing DOACs vs. standard therapy (EINSTEIN, AMPLIFY, Hokusai-VTE) was very different. The mean age was 55–57 years, 33–43% were women, and only 2.5–9% had active cancer [31,32,33]. As a result, studies performed in real-life conditions are important to ascertain whether the results of these clinical trials can be extended to the whole population taking FXai [10]. For example, in the case of the AF population, XANTUS (rivaroxaban), ETNA-AF Europe (edoxaban), and a retrospective study using four large US claims databases: MarketScan, PharMetrics, Optum, and Humana (apixaban), showed a similar clinical profile compared to our study, but in contrast to our data, these studies had a very limited follow-up [34,35,36]. On the other hand, in the RIETE registry, which included patients with VTE in Spain, those patients taking DOACs were older and had more comorbidities than those reported in the phase 3 clinical trials [37]. Therefore, our data are very relevant, since they may provide current information about patients taking FXai in Spain and the real incidence of bleeding in clinical practice.

In our study, the overall incidence rates of major, gastrointestinal, intracranial, and fatal bleedings at the end of the follow-up period were 10.2, 9.0, 0.8, and 0.5 per 100 person-years, respectively (10.1, 9.0, 0.7, and 0.4 per 100 person-years among those patients taking DOACs, respectively). In the case of the phase 3 clinical trials with DOACs in the AF population, these numbers were 2.1–3.6, 0.76–2.0, 0.33–0.49, and 0.2–0.21 per 100 person-years, respectively, in the active groups [28,29,30]. Although the classification of major bleeding was different to our study, as in the phase 3 clinical trials major bleeding was defined according to the International Society on Thrombosis and Haemostasis criteria [38], and we used a less restrictive definition, major bleeding rates were higher in our study, and intracranial and fatal bleedings had lower incidences. However, despite a lower incidence of major bleeding also being observed in other real-life studies compared to our data, no relevant differences have been shown regarding intracranial and fatal bleedings [34,35,36]. In summary, in clinical practice, the use of FXai is safe, even in high-risk patients, including the elderly population, with many comorbidities. Furthermore, in our study, the incidence of all types of bleeding decreased over time, suggesting that at anticoagulation initiation, predisposing conditions may play a larger role in bleeding risk rather than anticoagulation itself.

On the other hand, it has been shown that despite the increased risk of stroke and bleeding with age in patients with AF, the net clinical benefit clearly favors anticoagulation [39]. In our study, not only was increasing age associated with a higher risk of develoIping major bleeding, but also discontinuation of FXai after a major bleeding event. In addition, dose reduction of DOACs did not reduce the risk of major bleeding. Since prescribing inappropriate doses of DOACs, particularly underdosing, has been associated with a higher risk of events [40,41], our data emphasize the need to prescribe DOACs properly according to the product label [21,22,23]. In fact, in our study, in around 84% of patients, dosing of DOACs was performed adequately, and good compliance was achieved in nearly 92% of patients, although 65.7% of patients with major bleeding discontinued anticoagulation after the event, and this could have had an impact on the results. However, once the cause of bleeding is resolved, treatment with DOACs should be reintroduced promptly [16].

Although the incidence rates of major bleeding were higher than those reported in other studies, the incidence rates of fatal bleeding were low. In fact, among patients taking direct FXai, only 4% of major bleeding led to death, with 2.2% in the case of major gastrointestinal bleeding and 28.1% after an intracranial bleeding (vs. 15.4%, 5.6%, and 42.9% in those patients taking enoxaparin, respectively). In addition, when rebleeding occurred, the majority of cases had gastrointestinal origins. Therefore, although in the majority of cases, supportive measures combinedwith correcting the cause are sufficient for managing major bleeding, in some cases, particularly those with intracranial bleeding, reversal strategies are necessary [42,43]. In fact, mortality from ICH associated with the use of DOACs remained high, only slightly lower than that related to the use of VKA, even with the current reversal agents (prothrombin complex, basically). In this context, the use of DOAC-specific reversal agents is the best option [2,3]. Thus, andexanet Alpha has been demonstrated to rapidly restore coagulation in patients taking direct FXai to a greater extent than other alternatives [44].

Finally, rates of bleeding were much higher in the enoxaparin group. Due to limited evidence at the time of the study about the treatment with DOACs among patients with cancer, enoxaparin has traditionally been used as anticoagulant treatment in this population in Spain. As a result, we specifically analyzed this group of patients. However, considering that clinical trials have shown a better benefit–risk profile with oral direct FXai than with standard therapy, and the lack of evidence with enoxaparin for the prevention of stroke in AF patients [28,29,30], in light of our data, it would be preferable to use DOACs in clinical practice for patients with active cancer at risk of thromboembolic complications, rather than continuing with enoxaparin. In fact, an increase in the use of DOACs in this clinical setting is expected [17].

Our study has some limitations. First, as baseline variables were collected retrospectively, some relevant data could be missing. However, the large number of patients included could reduce this potential bias. In addition, the lack of a control group prevents direct comparisons from being performed. Although some antidepressants may have relevant interactions with DOACs, only the total number of antidepressants was recorded. Additionally, although some types of cancer may increase the risk of bleeding, only the total proportion of patients with cancer was collected. Furthermore, not all potent CYP-3A4 and P-pg inhibitors, which may interact with DOACs, were recorded. However, as the overall bleeding incidence or event rates did not significantly change according to the dosage of DOACs, the impact of the use of these drugs on our study may be limited. Finally, our data can only be extended to populations with a similar clinical profile and healthcare system.

In conclusion, in Spain, patients taking FXai were old and had many comorbidities. Despite higher incidence rates of major bleeding compared to those reported in clinical trials, partially due to differences in the clinical profile of patients and the definition of major bleeding, incidence rates of intracranial and fatal bleedings were low. Importantly, the availability of a specific antidote could help reduce this risk even more. As a result, FXai can be safely used in routine practice, but more efforts are required to reduce the risk of intracranial and fatal bleedings, due to their relevant clinical impact.

## Figures and Tables

**Table 1:** Baseline clinical characteristics at the index date (day 1 of treatment) and FXai use during the study period in the overall study population and according to the development of MB during the follow-up.

	Overall FXai Group N = 9374	DOACs				Enoxaparin (Active Oncology Patients Only)			
		All DOACs	Patients without MB in FU	Patients with a MB Event in FU		Enoxaparin per Bleeding Status			
		Overall (N = 8972)	Overall (N = 8502)	Overall (N = 470)	*p* (MB vs. No MB)	All Enoxaparin (N = 402)	Patients without MB in FU (N = 376)	Patients with MB in FU (N = 26)	*p* (MB vs. No MB)
Biodemographic data									
Age, years (SD)	71.8 (9.4)	71.8 (9.4)	71.5 (9.3)	77.4 (9.62)	0.07	72.2 (9.6)	72.0 (9.4)	75.9 (11.0)	0.002
<45 years, n (%)	42 (0.5)	39 (0.4)	39 (0.5)	0		3 (0.8)	3 (0.8)	0	
45–64 years, n (%)	2148 (22.9)	2063 (23.0)	2016 (23.7)	47 (10)		85 (21.1)	81 (21.5)	4 (15.4)	
65–74 years, n (%)	3722 (39.7)	3567 (39.8)	3428 (40.3)	139 (29.6)	<0.001	155 (38.6)	145 (38.6)	10 (38.5)	0.56
75–84 years, n (%)	2725 (29.1)	2599 (29.0)	2414 (28.4)	185 (39.4)		126 (31.3)	120 (31.9)	6 (23.1)	
≥85 years, n (%)	737 (7.9)	704 (7.9)	605 (7.1)	99 (21.1)		33 (8.2)	27 (7.2)	6 (23.1)	
Sex (female), n (%)	5247 (56.0)	5067 (56.5)	4780 (56.2)	287 (61.1)		180 (44.8)	169 (45.0)	11 (42.3)	
BMI, Kg/m^2^ (SD)	28.4 (5.3)	28.5 (5.3)	28.5 (5.3)	28.2 (5.3)	0.89	27.2 (4.3)	27.1 (4.3)	28.5 (4.4)	0.28
Alcohol use, n (%)	432 (4.6)	423 (4.7)	397 (4.7)	26 (5.5)	0.57	9 (2.2)	9 (2.4)	0	-
Updated Charlson index (SD)	6.3 (2.1)	6.2 (2.0)	6.2 (2.0)	6.1 (1.9)	0.96	9.4 (3.1)	9.4 (3.2)	9.2 (2.9)	0.84
Cardiovascular risk factors									
Hypertension, n (%)	7149 (76.3)	6865 (76.5)	6495 (76.4)	370 (78.7)	0.30	284 (70.7)	264 (70.2)	20 (76.9)	0.53
Hypercholesterolemia, n (%)	4087 (43.6)	3918 (43.7)	3701 (43.5)	217 (46.2)	0.45	169 (42.0)	161 (42.8)	8 (30.8)	0.50
Diabetes type 2, n (%)	3146 (33.6)	3011 (33.6)	2857 (33.6)	154 (32.8)	0.83	135 (33.6)	130 (34.6)	5 (19.2)	0.48
Diabetes type 1, n (%)	186 (2.0)	179 (2.0)	166 (2.0)	13 (2.8)	0.84	7 (1.7)	6 (1.6)	1 (3.9)	0.87
Smoking, n (%)	773 (8.3)	756 (8.4)	711 (8.4)	45 (9.6)	0.93	17 (4.3)	16 (4.3)	1 (3.9)	-
Vascular disease									
Heart failure, n (%)	2393 (25.5)	2286 (25.5)	2175 (25.6)	111 (23.6)	0.64	107 (26.6)	98 (26.1)	9 (34.6)	0.58
Chronic kidney disease, n (%)	1485 (15.8)	1430 (15.9)	1355 (15.9)	75 (16.0)	0.97	55 (13.7)	52 (13.8)	3 (11.5)	-
Coronary artery disease, n (%)	1452 (15.5)	1398 (15.6)	1328 (15.6)	70 (14.9)	0.87	54 (13.4)	53 (14.1)	1 (3.9)	0.77
Myocardial infarction, n (%)	660 (7.0)	637 (7.1)	604 (7.1)	33 (7.0)	0.99	23 (5.7)	23 (6.1)	0	-
Peripheral vascular disease, n (%)	721 (7.7)	692 (7.7)	651 (7.7)	41 (8.7)	0.81	29 (7.2)	27 (7.2)	2 (7.7)	0.98
Cerebrovascular disease, n (%)	600 (6.4)	569 (6.3)	536 (6.3)	33 (7.0)	0.87	31 (7.7)	30 (8.0)	1 (3.9)	0.88
Other comorbidities									
Chronic pulmonary disease, n (%)	1937 (20.7)	1865 (20.8)	1773 (20.9)	92 (19.6)	0.77	72 (17.9)	66 (17.6)	6 (23.1)	0.74
Cancer, n (%)	1190 (12.7)	788 (8.8)	743 (8.7)	45 (9.6)	0.85	402 (100)	376 (100)	26 (100)	-
Anemia, n (%)	994 (10.6)	946 (10.5)	897 (10.6)	49 (10.4)	0.98	48 (11.9)	46 (12.2)	2 (7.7)	0.85
MB history >60 days prior to FXai start, n (%)	886 (9.5)	855 (9.5)	805 (9.5)	50 (10.6)	0.79	31 (7.7)	26 (6.9)	5 (19.2)	0.34
Liver disease, n (%)	506 (5.4)	480 (5.4)	460 (5.4)	20 (4.3)	0.99	26 (6.5)	26 (6.91%)	0	-
Peptic ulcer disease, n (%)	319 (3.4)	311 (3.5)	291 (3.4)	20 (4.3)	0.84	8 (2.0)	8 (2.1)	0	-
FXai use during the follow-up									
Year of first FXai use, n (%)					0.89				0.99
2013	263 (2.8)	223 (2.5)	217 (2.6)	6 (1.3)		40 (10.0)	37 (9.8)	3 (11.5)	
2014	349 (3.7)	315 (3.5)	299 (3.5)	16 (3.4)		34 (8.5)	34 (9.0)	0	
2015	394 (4.2)	364 (4.1)	346 (4.1)	18 (3.8)		30 (7.5)	27 (7.2)	3 (11.5)	
2016	483 (5.2)	456 (5.1)	444 (5.2)	12 (2.6)		27 (6.7)	25 (6.7)	2 (7.7)	
2017	650 (6.9)	614 (6.8)	581 (6.8)	33 (7.0)		36 (9.0)	33 (8.8)	3 (11.5)	
2018	887 (9.5)	849 (9.5)	796 (9.4)	53 (11.3)		38 (9.5)	35 (9.3)	3 (11.5)	
2019	897 (9.6)	847 (9.4)	797 (9.4)	50 (10.6)		50 (12.4)	45 (12.0)	5 (19.2)	
2020	1248 (13.3)	1201 (13.4)	1138 (13.4)	63 (13.4)		47 (11.7)	44 (11.7)	3 (11.5)	
2021	2050 (21.9)	2005 (22.4)	1905 (22.4)	100 (21.3)		45 (11.2)	44 (11.7)	1 (3.9)	
2022	2153 (23.0)	2098 (23.4)	1979 (23.3)	119 (25.3)		55 (13.7)	52 (13.8)	3 (11.5)	
FXai indications, n (%)					0.36				<0.001
VTE	2080 (22.2)	1932 (21.5)	1849 (21.8)	83 (17.7)		148 (36.8)	138 (36.7)	10 (38.5)	
AF	6773 (72.3)	6547 (78.0)	6180 (72.7)	367 (78.1)		226 (56.2)	213 (56.7)	13 (50.0)	
Non-mechanical cardiac-valve replacement	521 (5.6)	493 (5.5)	473 (5.6)	20 (4.3)		28 (7.0)	25 (6.7)	3 (11.5)	
Dose as per label, n (%)					0.94				0.56
No dose reduction	5152 (55.0)	4886 (54.5)	4641 (54.6)	245 (52.1)		266 (66.2)	244 (64.9)	22 (84.6)	
Dose reduction as per label	2764 (29.5)	2661 (29.7)	2520 (29.6)	141 (30.0)		103 (25.6)	100 (26.6)	3 (11.5)	
Dose higher or lower than label	1458 (15.6)	1425 (15.9)	1341 (15.8)	84 (17.9)		33 (8.2)	32 (8.5)	1 (3.9)	
Medical possesion ratio to FXai (% prescribed doses taken) ^1^	86.4 (4.6)	86.3 (4.7)	86.3 (4.7)	86.0 (4.7)	0.97	87.5 (2.8)	87.6 (2.8)	86.8 (2.1)	0.87
Compliance ≥80%, n (%)	8592 (91.7)	8193 (91.3)	7769 (91.4)	424 (90.2)	0.41	399 (99.3)	373 (99.)	26 (100)	0.65
Discontinuation after 1st MB, n (%)	327 (3.5)	309 (3.4)	0	309 (65.7)	-	18 (4.5)	0	18 (69.2)	-
Concomitant treatments (within 120 days prior to index)									
Gastroprotective drugs, n (%)	6504 (69.4)	6205 (69.2)	5884 (69.2)	321 (68.3)	0.73	299 (74.4)	279 (74.2)	20 (76.9)	0.79
Antihypertensive therapies, n (%)	5752 (61.4)	5524 (61.6)	5221 (61.4)	303 (64.5)	0.29	228 (56.7)	211 (56.1)	17 (65.4)	0.46
Lipid lowering drugs, n (%)	5145 (54.9)	4949 (55.2)	4677 (55.0)	272 (57.9)	0.36	196 (48.8)	183 (48.7)	13 (50.0)	0.93
Antidiabetic drugs, n (%)	2854 (30.5)	2737 (30.5)	2591 (30.5)	146 (31.1)	0.98	117 (29.1)	114 (30.3)	3 (11.5)	0.99
NSAIDs, n (%)	2821 (30.1)	2698 (30.1)	2541 (29.9)	157 (33.4)	0.35	123 (30.6)	117 (31.1)	6 (23.1)	0.68
Antiplatelet drugs, n (%)	1457 (15.5)	1391 (15.5)	1323 (15.6)	68 (14.5)	0.81	66 (16.4)	61 (16.2)	5 (19.2)	0.86
Acetylsalicylic acid, n (%)	1419 (15.1)	1354 (15.1)	1290 (15.2)	64 (13.6)	0.74	65 (16.2)	60 (16.0)	5 (19.2)	0.85
Acetylsalicylic acid, combinations with proton pump inhibitors, n (%)	46 (0.5)	45 (0.5)	41 (0.5)	4 (0.9)	0.92	1 (0.3)	1 (0.3)	0	-
Antidepressants, n (%)	878 (9.4)	835 (9.3)	793 (9.3)	42 (8.9)	0.93	43 (10.7)	40 (10.6)	3 (11.5)	0.96
Anticancer drugs *, n (%)	885 (9.4)	602 (6.7)	568 (6.7)	34 (7.2)	0.90	283 (70.4)	263 (70.0)	20 (76.9)	0.51
Biochemical parameters									
Hemoglobin, g/dL (SD)	12.1 (3.2)	12.1 (3.2)	12.1 (3.2)	11.8 (3.3)	0.87	11.8 (3.2)	11.8 (3.2)	11.2 (3.4)	0.61
HbA1c, % (SD)	7.4 (1.2)	7.4 (1.2)	7.43 (1.2)	7.36 (1.1)	0.98	7.2 (1.3)	7.2 (1.2)	7.3 (1.4)	0.94
Platelet count, ×10^3^/µL	276.9 (144.7)	277.4 (144.3)	277.4 (144.5)	276.7 (140.0)	0.62	265.7 (152.4)	264.4 (152.3)	284.3 (155.5)	<0.001
eGFR, mL/min/1.73 m^2^ (SD)	94.7 (11.2)	94.7 (11.2)	94.7 (11.1)	94.5 (12.1)	0.96	94.3 (11.3)	94.3 (10.8)	94.7 (16.9)	0.77
Creatinine clearance, mL/min (SD)	50.8 (22.5)	50.8 (22.3)	50.9 (22.4)	48.6 (22.1)	0.12	50.4 (24.7)	50.6 (24.7)	48.0 (24.4)	0.06

^1^ Consider: two tablets or capsules per day for apixaban and one per day for rivaroxaban and edoxaban; * within 180 days prior to the index date. AF: atrial fibrillation; BMI: body mass index; DOACs: direct oral anticoagulants; eGFR: estimated glomerular filtration rate; FXai; Factor Xa inhibitors; FU: follow-up; MB: major bleeding; NSAIDs: non-steroidal anti-inflammatory drugs; SD: standard deviation; VTE: venous thromboembolism.

## Data Availability

This was a secondary data study using the BIG-PAC® database, and the data can be obtained upon reasonable request.

## Supplementary Materials

The following supporting information can be downloaded at: https://www.mdpi.com/article/10.3390/jcm13061677/s1, Table S1. Major bleeding: all critical site bleedings and anemia codes (ICD-9 and ICD-10 classifications); other bleeding codes were considered major only if they were fatal or caused hospitalization. Table S2. Therapeutic doses of direct oral anticoagulants and enoxaparin. Table S3. Baseline clinical characteristics according to the FXai indication at first prescription and the specific DOAC. Table S4. CHA_2_D_2_-VASC and HASBLED scores at index date and at 3 months from index date. Table S5. Major bleeding. Table S6. Major gastrointestinal bleeding. Table S7. Intracranial bleeding. Table S8. Trauma-related bleeding event and incidence rates. Table S9. Other major bleeding event and incidence rates. Table S10. Risk factors for major bleeds using COX regression models for DOAC patients.
