# Supplementary material for: Clinical Characteristics and Incidence of Hemorrhagic Complications in Patients Taking Factor Xa Inhibitors in Spain: A Long-Term Observational Study
Source: J Clin Med. 2024 Mar 14;13(6):1677. doi: 10.3390/jcm13061677 (PMC10970821; doi:10.3390/jcm13061677)
Supplement: Supplementary file 1 [file jcm-13-01677-s001.zip › jcm-2898293-supplementary.pdf]

Supplementary Table S1. Major bleeding: all critical site bleedings and anemia codes (ICD-9 and ICD-10 classifications); other bleeding codes were considered major only if they were fatal or caused hospitalization.

ICD-10	Diagnosis	MB classification	Subtype	Comments
D699	Hemorrhagic condition, unspecified	Other	Unspecified	ONLY CONSIDERED MAJOR IF FATAL BLEEDING OR CAUSING HOSPITALIZATION
J942	Hemothorax	Other	Respiratory	
K226	Gastro-esophageal laceration-hemorrhage syndrome	GIB	Upper	
K250	Gastric ulcer; Acute with hemorrhage	GIB	Upper	
K252	Gastric ulcer; Acute with both hemorrhage and perforation	GIB	Upper	
K254	Gastric ulcer; Chronic or unspecified with hemorrhage	GIB	Upper	
K256	Gastric ulcer; Chronic or unspecified with both hemorrhage and perforation	GIB	Upper	
K260	Duodenal ulcer; Acute with hemorrhage	GIB	Upper	
K262	Duodenal ulcer; Acute with both hemorrhage and perforation	GIB	Upper	
K264	Duodenal ulcer; Chronic or unspecified with hemorrhage	GIB	Upper	
K266	Duodenal ulcer; Chronic or unspecified with both hemorrhage and perforation	GIB	Upper	
K270	Peptic ulcer, site unspecified; Acute with hemorrhage	GIB	Upper	
K272	Peptic ulcer, site unspecified; Acute with both hemorrhage and perforation	GIB	Upper	
K274	Peptic ulcer, site unspecified; Chronic or unspecified with hemorrhage	GIB	Upper	
K276	Peptic ulcer, site unspecified; Chronic or unspecified with both hemorrhage and perforation	GIB	Upper	
K280	Gastrojejunal ulcer; Acute with hemorrhage	GIB	Upper	
K282	Gastrojejunal ulcer; Acute with both hemorrhage and perforation	GIB	Upper	
K284	Gastrojejunal ulcer; Chronic or unspecified with hemorrhage	GIB	Upper	
K286	Gastrojejunal ulcer; Chronic or unspecified with both hemorrhage and perforation	GIB	Upper	
K290	Acute hemorrhagic gastritis	GIB	Upper	
K625	Hemorrhage of anus and rectum	GIB	Lower	
K661	Hemoperitoneum	Other	Unspecified	
K920	Hematemesis	GIB	Lower	
K921	Melena	GIB	Lower	
K922	Gastrointestinal hemorrhage, unspecified	GIB	Unspecified	
N020	Recurrent and persistent hematuria; Minor glomerular abnormality	Other	Genitourinary	
N021	Recurrent and persistent hematuria; Focal and segmental glomerular lesions	Other	Genitourinary	
N022	Recurrent and persistent hematuria; Diffuse membranous glomerulonephritis	Other	Genitourinary	
N023	Recurrent and persistent hematuria; Diffuse mesangial proliferative glomerulonephritis	Other	Genitourinary	
N024	Recurrent and persistent hematuria; Diffuse endocapillary proliferative glomerulonephritis	Other	Genitourinary	
N025	Recurrent and persistent hematuria; Diffuse mesangiocapillary glomerulonephritis	Other	Genitourinary	
N026	Recurrent and persistent hematuria; Dense deposit disease	Other	Genitourinary	
N027	Recurrent and persistent hematuria; Diffuse crescentic glomerulonephritis	Other	Genitourinary	
N028	Recurrent and persistent hematuria; Other	Other	Genitourinary	
N029	Recurrent and persistent hematuria; Unspecified	Other	Genitourinary	
N421	Congestion and hemorrhage of prostate	Other	Genitourinary	
N836	Hematosalpinx	Other	Genitourinary	
N837	Hematoma of broad ligament	Other	Genitourinary	
N857	Hematometra	Other	Genitourinary	
N897	Hematocolpos	Other	Genitourinary	
N920	Excessive and frequent menstruation with regular cycle	Other	Genitourinary	
N921	Excessive and frequent menstruation with irregular cycle	Other	Genitourinary	
N922	Excessive menstruation at puberty	Other	Genitourinary	
N923	Ovulation bleeding	Other	Genitourinary	

N924	Excessive bleeding in the premenopausal period	Other	Genitourinary	
N930	Postcoital and contact bleeding	Other	Genitourinary	
N938	Other specified abnormal uterine and vaginal bleeding	Other	Genitourinary	
N939	Abnormal uterine and vaginal bleeding, unspecified	Other	Genitourinary	
N950	Postmenopausal bleeding	Other	Genitourinary	
R040	Epistaxis	Other	Respiratory	
R041	Hemorrhage from throat	Other	Respiratory	
R042	Hemoptysis	Other	Respiratory	
R048	Hemorrhage from other sites in respiratory passages	Other	Respiratory	
R049	Hemorrhage from respiratory passages, unspecified	Other	Respiratory	
R31	Unspecified hematuria	Other	Genitourinary	
R58	Hemorrhage, not elsewhere classified	Other	Unspecified	
S271	Traumatic hemothorax	Other	Respiratory	
T792	Traumatic secondary and recurrent hemorrhage	Other	Unspecified	
T810	Hemorrhage and hematoma complicating a procedure, not elsewhere classified	Other	Unspecified	
**CRITICAL SITE: ALWAYS MAJOR: all lines below, including intraocular bleedings and anemia codes**				
I230	Hemopericardium as current complication following acute myocardial infarction	Other	Hemopericardium	ALWAYS CONSIDERED MAJOR
I312	Hemopericardium, not elsewhere classified	Other	Hemopericardium	
I600	Subarachnoid hemorrhage from carotid siphon and bifurcation	ICH	SAH	
I601	Subarachnoid hemorrhage from middle cerebral artery	ICH	SAH	
I602	Subarachnoid hemorrhage from anterior communicating artery	ICH	SAH	
I603	Subarachnoid hemorrhage from posterior communicating artery	ICH	SAH	
I604	Subarachnoid hemorrhage from basilar artery	ICH	SAH	
I605	Subarachnoid hemorrhage from vertebral artery	ICH	SAH	
I606	Subarachnoid hemorrhage from other intracranial arteries	ICH	SAH	
I607	Subarachnoid hemorrhage from intracranial artery, unspecified	ICH	SAH	
I608	Other subarachnoid hemorrhage	ICH	SAH	
I609	Subarachnoid hemorrhage, unspecified	ICH	SAH	
I610	Intracerebral hemorrhage in hemisphere, subcortical	ICH	ICH	
I611	Intracerebral hemorrhage in hemisphere, cortical	ICH	ICH	
I612	Intracerebral hemorrhage in hemisphere, unspecified	ICH	ICH	
I613	Intracerebral hemorrhage in brain stem	ICH	ICH	
I614	Intracerebral hemorrhage in cerebellum	ICH	ICH	
I615	Intracerebral hemorrhage, intraventricular	ICH	ICH	
I616	Intracerebral hemorrhage, multiple localized	ICH	ICH	
I618	Other intracerebral hemorrhage	ICH	ICH	
I619	Intracerebral hemorrhage, unspecified	ICH	ICH	
I620	Subdural hemorrhage (acute)(nontraumatic)	ICH	Other ICH	
I621	Nontraumatic extradural hemorrhage	ICH	Other ICH	
I629	Intracranial hemorrhage (nontraumatic), unspecified	ICH	Other ICH	
I713	Abdominal aortic aneurysm, ruptured	Other	Retroperitoneal	
I715	Thoracoabdominal aortic aneurysm, ruptured	Other	Unspecified	
I718	Aortic aneurysm of unspecified site, ruptured	Other	Unspecified	
M250	Hemarthrosis	Other	Hemarthrosis	
P544	Neonatal adrenal hemorrhage	Other	Retroperitoneal	
S064	Epidural hemorrhage	ICH	Other ICH	
S065	Traumatic subdural hemorrhage	ICH	Other ICH	
S066	Traumatic subarachnoid hemorrhage	ICH	SAH	
	Critical intra-ocular bleeding: 4 lines below	Other	Unspecified	

H313	-Choroidal hemorrhage and rupture	Other	Unspecified	
H356	Retinal hemorrhage	Other	Unspecified	
H431	Vitreous hemorrhage	Other	Unspecified	
H450	Vitreous hemorrhage in diseases classified elsewhere	Other	Unspecified	
**Anemia: 2 lines below –** If in temporal relationship wth a major bleeding location code, the location code was used instead				
D500	Iron deficiency anemia secondary to blood loss (chronic)	Other	Unspecified	ALWAYS CONSIDERED MAJOR
D62	Acute posthemorrhagic anemia	Other	Unspecified	

GIB: gastrointestinal bleeding; ICD: International Classification of Diseases; ICH: intracranial hemorrhage; MB: Major Bleeding; SAH: subarachnoid hemorrhage.

ICD-9	diagnosis	MB Classification	Subtype
280	Anemia due to blood loss	Other	Unspecified
285,1	Acute posthemorrhagic anemia	Other	Unspecified
285,9	Anemia, unspecified	Other	Unspecified
430	Subarachnoid hemorrhage	ICH	SAH
431	Intracerebral hemorrhage	ICH	ICH
432,1	Subdural hemorrhage	ICH	Other
455	Hemorrhoids	GIB	Lower
455,2	Internal hemorrhoids with other complication	GIB	Lower
455,8	Unspecified hemorrhoids with other complication	GIB	Lower
459	Hemorrhage, unspecified	Other	Unspecified
530,1	Esophagitis	GIB	Upper
530,7	Mallory-Weiss tear	GIB	Upper
531.0x	Acute gastric ulcer with hemorrhage	GIB	Upper
531.4x	Chronic or unspecified gastric ulcer with hemorrhage	GIB	Upper
532.0x	Acute duodenal ulcer with hemorrhage	GIB	Upper
532.4x	Chronic or unspecified duodenal ulcer with hemorrhage	GIB	Upper
533.0x	Acute peptic ulcer, site unspecified, hemorrhage	GIB	Upper
535,01	Acute gastritis with hemorrhage	GIB	Upper
535,11	Atrophic gastritis with hemorrhage	GIB	Upper
535,41	Other specified gastritis with hemorrhage	GIB	Upper
535,51	Unspecified gastritis and gastroduodenitis with hemorrhage	GIB	Upper
535,61	Duodenitis with hemorrhage	GIB	Upper
537,83	Angiodysplasia of stomach and duodenum with hemorrhage	GIB	Upper
562,1	Diverticula of colon without mention of hemorrhage	GIB	Lower
562,13	Diverticulosis of colon with hemorrhage	GIB	Lower
562,13	Diverticulitis of colon with hemorrhage	GIB	Lower
568,81	Hemoperitoneum	Other	Unspecified
569,3	Hemorrhage of rectum and anus	GIB	Lower
569,85	Angiodysplasia of intestine with hemorrhage	GIB	Lower
578	Hematemesis	GIB	Upper
578,1	Blood in stool	GIB	Lower
578,9	Hemorrhage of gastrointestinal tract, unspecified	GIB	Unspecified
599,7	Hematuria	Other	Genitourinary
623,8	Other specified noninflammatory disorders of vagina	Other	Genitourinary
626,2	Excessive/frequent menstruation	Other	Genitourinary
719.1x	Hemarthrosis	Other	Hemarthrosis
784,7	Epistaxis	Other	Respiratory
796,3	Hemoptysis	Other	Respiratory
790,92	Abnormal coagulation profile	Other	Other

GIB: gastrointestinal bleeding; ICD: International Classification of Diseases; ICH: intracranial hemorrhage; MB: Major Bleeding.

Supplementary Table S2. Therapeutic doses of direct oral anticoagulants and enoxaparin.

Apixaban	Prevention of VTE in adult patients who have undergone elective hip or knee replacement surgery	2.5 mg/12 hs. The initial dose should be taken within 12-24 hours after surgery.
	Stroke prevention (NVAF)	Standard dose: 5 mg/12 hs<br>Adjusted dose: 2.5 mg/12 hs, if creatinine clearance 15-29 ml/min or ≥2 of the following conditions: age ≥ 80 years, body weight ≤ 60 kg, or serum creatinine ≥ 1.5 mg/dl (133 mmol/l).
	Treatment of DVT and PE	10 mg/12 hs during the first 7 days, followed by 5 mg/12 hs
	Prevention of recurrences of DVT and/or PE after completion of treatment of DVT or PE for 6 months	2.5 mg/12 hs
Edoxaban	Stroke prevention (NVAF)	Standard dose: 60 mg once daily<br>Adjusted dose: 30 mg once daily if creatinine clearance 15-50 ml/min, or body weight ≤ 60 kg or concomitant treatment with P-gp inhibitors (ciclosporin, dronedarone, erythromycin, ketoconazole).
	Treatment of DVT and PE (start treatment after initial parenteral anticoagulation at least during 5 days)	Standard dose: 60 mg once daily at least 5 days<br>Adjusted dose: 30 mg once daily if creatinine clearance 15-50 ml/min, or body weight ≤ 60 kg or concomitant treatment with P-gp inhibitors (ciclosporin, dronedarone, erythromycin, ketoconazole).
	Prevention of recurrences of DVT and/or PE after completion of treatment of DVT or PE for 6 months	Standard dose: 60 mg once daily<br>Adjusted dose: 30 mg once daily if creatinine clearance 15-50 ml/min, or body weight ≤ 60 kg or concomitant treatment with P-gp inhibitors (ciclosporin, dronedarone, erythromycin, ketoconazole).
Rivaroxaban	Stroke prevention (NVAF)	Standard dose: 20 mg once daily<br>Adjusted dose: 15 mg once daily if creatinine clearance 15-50 ml/min
	Treatment of DVT and PE	Day 1-21: 15 mg/12 hs<br>Day 22 onwards: 20 mg once daily (15 mg once daily if high bleeding risk)
	Prevention of recurrences of DVT and/or PE in adults	Following completion of at least 6 months therapy for DVT or PE: 10 once daily or 20 mg once daily if high risk of DVT or PE or event while taking 10 mg
	Prevention VTE in adult patients who have undergone elective hip or knee replacement surgery	10 mg of rivaroxaban once daily. The initial dose should be taken within 6-10 hours after surgery. After knee replacement surgery, for 2 weeks; after hip replacement surgery, for 5 weeks.
Enoxaparin	Only active cancer patients with at least one of the following indications:<br>- Venous thromboembolism<br>- AF<br>- Non-mechanical cardiac-valve replacement	Anticoagulant doses:<br>1 mg/kg/12 hs<br>≥100 mg/24 hs<br>Severe renal disease: 1 mg/kg/day

DVT: deep vein thrombosis; hs: hours; NVAF: nonvalvular atrial fibrillation; PE: pulmonary embolism; VTE: venous thromboembolic events.

Supplementary Table S3. Baseline clinical characteristics according to the FXai indication at first prescription and the specific DOAC.

	Overall FXai group N=9374	DOACs							Enoxaparin (active oncology patients only)			
		All DOACs	Specific DOAC			DOAC indication at first prescription			All enoxaparin (N=402)	Enoxaparin indication at first prescription		
		Overall (N=8972)	Apixaban (N=4441)	Rivaroxaban (N=2355)	Edoxaban (N=2176)	VTE (N=1932)	AF (N=6547)	Non-mechanical cardiac-valve replacement (N=493)		VTE (N=148)	AF (N=226)	Non-mechanical cardiac-valve replacement (N=28)
**Biodemographic data**												
Age, years (SD)	71.8 (9.4)	71.8 (9.4)	71.9 (9.5)	71.6 (9.4)	71.7 (9.2)	71.7 (9.4)	71.8 (9.4)	71.4 (10.1)	72.2 (9.6)	72.0 (10.5)	72.7 (8.7)	70.0 (11.3)
<45 years, n (%)	42 (0.5)	39 (0.43%)	19 (0.4)	12 (0.5)	8 (0.4)	6 (0.3)	33 (0.5)	0	3 (0.8)	2 (1.4)	0	1 (3.6)
45-64 years, n (%)	2148 (22.9)	2063 (23.0)	1012 (22.8)	555 (23.6)	496 (22.8)	440 (22.8)	1490 (22.8)	133 (27.0)	85 (21.1)	36 (24.3)	42 (18.6)	7 (25.0)
65-74 years, n (%)	3722 (39.7)	3567 (39.8)	1769 (39.8)	922 (39.2)	876 (40.3)	782 (40.5)	2605 (39.8)	180 (36.5)	155 (38.6)	49 (33.1)	95 (42.0)	11 (39.3)
75-84 years, n (%)	2725 (29.1)	2599 (29.0)	1268 (28.6)	689 (29.3)	642 (29.5)	550 (28.5)	1918 (29.3)	131 (26.6)	126 (31.3)	49 (33.1)	70 (31.0)	7 (25.0)
≥85 years, n (%)	737 (7.9)	704 (7.9)	373 (8.4)	177 (7.5)	154 (7.1)	154 (8.0)	501 (7.7)	49 (9.9)	33 (8.2)	12 (8.1)	19 (8.4)	2 (7.1)
Sex (female), n (%)	5247 (56.0)	5067 (56.5)	2402 (54.1)	1422 (60.4)	1243 (57.1)	1094 (56.6)	3688 (56.3)	285 (57.8)	180 (44.8)	66 (44.6)	98 (43.4)	16 (57.1)
BMI, Kg/m^2 (SD)	28.4 (5.3)	28.5 (5.3)	29.1 (5.6)	27.9 (5.3)	27.9 (4.7)	28.5 (5.2)	28.5 (5.3)	28.7 (5.4)	27.2 (4.3)	27.5 (4.1)	27.2 (4.3)	25.8 (5.0)
Alcohol use, n (%)	432 (4.6)	423 (4.7)	185 (4.2)	127 (5.4)	111 (5.1)	105 (5.4)	296 (4.5)	22 (4.5)	9 (2.2)	6 (4.1)	0	3 (10.7)
CHA2DS2-VASc (SD)	3.3 (1.5)	3.3 (1.5)				3.3 (1.5)	3.3 (1.5)	3.5 (1.5)	3.1 (1.5)	2.8 (1.3)	3.1 (1.5)	3.9 (1.9)
HAS-BLED (SD)	1.5 (1.0)	1.5 (0.9)				1.5 (1.0)	1.5 (0.9)	1.4 (1.0)	1.4 (1.1)	1.4 (1.0)	1.4 (1.0)	1.8 (1.2)
Charlson index (SD)	6.3 (2.1)	6.2 (2.0)	6.2 (2.0)	6.4 (1.9)	6.01(2.0)	6.16 (2.0)	6.2 (2.0)	6.4 (1.9)	9.4 (3.1)	9.3 (3.1)	9.5 (3.1)	8.9 (3.6)
**Cardiovascular risk factors**												
Hypertension, n (%)	7149 (76.3)	6865 (76.5)	3398 (76.5)	1866 (79.2)	1601 (73.6)	1469 (76.0)	5014 (76.6)	382 (77.5)	284 (70.7)	101 (68.2)	162 (71.7)	21 (75.0)
Hypercholesterolemia, n (%)	4087 (43.6)	3918 (43.7)	1994 (44.9)	1035 (44.0)	889 (40.9)	837 (43.3)	2863 (43.7)	218 (44.2)	169 (42.0)	68 (46.0)	88 (38.9)	13 (46.4)
Diabetes type 2, n (%)	3146 (33.6)	3011 (33.6)	1495 (33.7)	831 (35.3)	685 (31.5)	634 (32.8)	2212 (33.8)	165 (33.5)	135 (33.6)	47 (31.8)	79 (35.0)	9 (32.1)
Diabetes type 1, n (%)	186 (2.0)	179 (2.0)	84 (1.9)	41 (1.7)	54 (2.5)	41 (2.1)	129 (2.0)	9 (1.8)	7 (1.7)	1 (0.7)	5 (2.2)	1 (3.6)
Smoking, n (%)	773 (8.3)	756 (8.4)	364 (8.2)	195 (8.3)	197 (9.1)	161 (8.3)	556 (8.5)	39 (7.9)	17 (4.3)	11 (7.4)	4 (1.8)	2 (7.1)
**Vascular disease**												
Heart failure, n (%)	2393 (25.5)	2286 (25.5)	1136 (25.6)	661 (28.1)	489 (22.5)	457 (23.7)	1527 (23.3)	302 (61.3)	107 (26.6)	28 (18.9)	60 (26.6)	19 (67.9)
Chronic kidney disease, n (%)	1485 (15.8)	1430 (15.9)	716 (16.1)	376 (16.0)	338 (15.5)	286 (14.8)	1068 (16.3)	76 (15.4)	55 (13.7)	18 (12.2)	29 (12.8)	8 (28.6)
Coronary artery disease, n (%)	1452 (15.5)	1398 (15.6)	713 (16.1)	377 (16.0)	308 (14.2)	283 (14.7)	1036 (15.8)	79 (16.0)	54 (13.4)	19 (12.8)	32 (14.2)	3 (10.7)
Myocardial infarction, n (%)	660 (7.0)	637 (7.1)	312 (7.0)	186 (7.9)	139 (6.4)	128 (6.6)	474 (7.2)	35 (7.1)	23 (5.7)	6 (4.1)	16 (7.1)	1 (3.6)
Peripheral vascular disease, n (%)	721 (7.7)	692 (7.7)	332 (7.5)	189 (8.0)	171 (7.9)	132 (6.8)	524 (8.0)	36 (7.3)	29 (7.2)	7 (4.7)	20 (8.9)	2 (7.1)
Cerebrovascular disease, n (%)	600 (6.4)	569 (6.3)	268 (6.0)	158 (6.7)	143 (6.6)	121 (6.3)	413 (6.3)	35 (7.1)	31 (7.7)	8 (5.4)	17 (7.5)	6 (21.4)
**Other comorbidities**												
Chronic pulmonary disease, n (%)	1937 (20.7)	1865 (20.8)	778 (17.5)	515 (21.9)	572 (26.3)	414 (21.4)	1352 (20.7)	99 (20.1)	72 (17.9)	26 (17.6)	44 (19.5)	2 (7.1)
Cancer, n (%)	1190 (12.7)	788 (8.8)	416 (9.4)	195 (8.3)	177 (8.1)	169 (8.8)	576 (8.8)	43 (8.7)	402 (100)	148 (100)	226 (100)	28 (100)
Anemia, n (%)	994 (10.6)	946 (10.5)	458 (10.3)	282 (12.0)	206 (9.5)	192 (9.9)	705 (10.8)	49 (9.9)	48 (11.9)	16 (10.8)	30 (13.3)	2 (7.1)
MB history >60 days prior to FXai start, n (%)	886 (9.5)	855 (9.5)	451 (10.2)	243 (10.3)	161 (7.4)	181 (9.4)	621 (9.5)	53 (10.8)	31 (7.7)	13 (8.8)	18 (8.0)	0
Liver disease, n (%)	506 (5.4)	480 (5.4)	243 (5.5)	135 (5.7)	102 (4.7)	100 (5.2)	352 (5.4%)	28 (5.7)	26 (6.5)	10 (6.8)	15 (6.64)	1 (3.6)
Peptic ulcer disease, n (%)	319 (3.4)	311 (3.5)	141 (3.2)	94 (4.0)	76 (3.5)	65 (3.4)	229 (3.5)	17 (3.5)	8 (2.0)	5 (3.4)	2 (0.9)	1 (3.6)
**FXai use**												
Year of first FXai use, n (%)												
2013	263 (2.8)	223 (2.5)	99 (2.2)	124 (5.3)	0	44 (2.3)	164 (2.5)	15 (3.0)	40 (10.0)	15 (10.1)	20 (8.9)	5 (17.9)
2014	349 (3.7)	315 (3.5)	183 (4.1)	132 (5.6)	0	65 (3.4)	232 (3.5)	18 (3.7)	34 (8.5)	12 (8.1)	20 (8.9)	2 (7.1)
2015	394 (4.2)	364 (4.1)	226 (5.1)	138 (5.9)	0	81 (4.2)	264 (4.0)	19 (3.9)	30 (7.5)	10 (6.8)	16 (7.1%)	4 (14.3)
2016	483 (5.2)	456 (5.1)	276 (6.2)	146 (6.2)	34 (1.6)	84 (4.4)	340 (5.2)	32 (6.5)	27 (6.7)	11 (7.4)	15 (6.6)	1 (3.6)
2017	650 (6.9)	614 (6.8)	270 (6.1)	185 (7.9)	159 (7.3)	136 (7.0)	449 (6.9)	29 (5.9)	36 (9.0)	16 (10.8)	18 (8.0)	2 (7.1)
2018	887 (9.5)	849 (9.5)	360 (8.1)	222 (9.4)	267 (12.3)	167 (8.6)	643 (9.8)	39 (7.9)	38 (9.5)	15 (10.1)	19 (8.4)	4 (14.3)
2019	897 (9.6)	847 (9.4)	362 (8.2)	219 (9.3)	266 (12.2)	189 (9.8)	610 (9.3)	48 (9.7)	50 (12.4)	23 (15.5)	24 (10.6)	3 (10.7)
2020	1248 (13.3)	1201 (13.4)	620 (14.0)	258 (11.0)	323 (14.8)	277 (14.3)	857 (13.1)	67 (13.6)	47 (11.7)	16 (10.8)	27 (12.0)	4 (14.3)
2021	2050 (21.9)	2005 (22.4)	992 (22.3)	458 (19.5)	555 (25.5)	403 (20.9)	1480 (22.6)	122 (24.8)	45 (11.2)	19 (12.8)	23 (10.2)	3 (10.7)
2022	2153 (23.0)	2098 (23.4)	1053 (23.7)	473 (20.1)	572 (26.3)	486 (25.2)	1508 (23.0)	104 (21.1)	55 (13.7)	11 (7.4)	44 (19.5)	0
FXai indications, n (%)												
VTE	2080 (22.2)	1932 (21.5)	867 (19.5)	480 (20.4)	585 (26.9)	1932 (100)	0	0	148 (36.8)	148 (100)	0	0
AF	6773 (72.3)	6547 (78.0)	3296 (74.2)	1707 (72.5)	1544 (71.0)	0	6547 (100)	0	226 (56.2)	0	226 (100)	0
Non-mechanical cardiac-valve replacement	521 (5.6)	493 (5.5)	278 (6.3)	168 (7.1)	47 (2.2)	0	0	493 (100)	28 (7.0)	0	0	28 (100)
DOAC dose as per label, n (%)												
No dose reduction	5152 (55.0)	4886 (54.5)	2460 (55.4)	1273 (54.1)	1153 (53.0)	1033 (53.5)	3584 (54.7)	269 (54.6)	266 (66.2)	100 (67.6)	148 (65.5)	18 (64.3)
Dose reduction as per label	2764 (29.5)	2661 (29.7)	1294 (29.1)	764 (32.4)	603 (27.7)	589 (30.5)	1927 (29.4)	145 (29.4)	103 (25.6)	36 (24.3)	58 (25.7)	9 (32.1)
Dose higher or lower than label	1458 (15.6)	1425 (15.9)	687 (15.5)	318 (13.5)	420 (19.3)	310 (16.1)	1036 (15.8)	79 (16.0)	33 (8.2)	12 (8.1)	20 (8.9)	1 (3.6)

Medical possesion ratio to FXai (% prescribed doses taken)[4]	86.4 (4.6)	86.3 (4.7)	86.3 (4.2)	87.4 (4.8)	85.0 (5.2)	86.3 (4.8)	86.3 (4.7)	86.7 (4.7)	87.5 (2.8)	87.9 (2.8)	87.3 (2.8)	87.3 (2.9)
Compliance ≥80%, n (%)	8592 (91.7)	8193 (91.3)	4147 (93.4)	2228 (94.6)	1818 (83.6)	1755 (90.8)	5987 (91.5)	451 (91.5)	399 (99.3)	148 (100)	224 (99.1)	27 (96.4)
Discontinuation after MB, n (%)	327 (3.5)	309 (3.4)	152 (3.4)	78 (3.3)	79 (3.6)	53 (2.7)	246 (3.8)	10 (2.0)	18 (4.5)	9 (6.1)	8 (3.5)	1 (3.6)
**Concomitant treatments (within 120 days prior to index)**												
Gastroprotective drugs, n (%)	6504 (69.4)	6205 (69.2)	3086 (69.5)	1634 (69.4)	1485 (68.2)	1295 (67.0)	4582 (70.0)	328 (66.5)	299 (74.4)	107 (72.3)	169 (74.8)	23 (82.1)
Antihypertensive therapies, n (%)	5752 (61.4)	5524 (61.6)	2780 (62.6)	1465 (62.2)	1279 (58.8)	1179 (61.0)	4049 (61.9)	296 (60.0)	228 (56.7)	80 (54.1)	129 (57.1)	19 (67.9)
Lipid lowering drugs, n (%)	5145 (54.9)	4949 (55.2)	2441 (55.0)	1323 (56.2)	1185 (54.5)	1058 (54.8)	3583 (54.7)	308 (62.5)	196 (48.8)	62 (41.9)	115 (50.9)	19 (67.9)
Antidiabetic drugs, n (%)	2854 (30.5)	2737 (30.5)	1355 (30.5)	750 (31.9)	632 (29.0)	580 (30.0)	2005 (30.6)	152 (30.8)	117 (29.1)	41 (27.7)	67 (29.7)	9 (32.1)
NSAIDs, n (%)	2821 (30.1)	2698 (30.1)	1299 (29.3)	728 (30.9)	671 (30.8)	593 (30.7)	1966 (30.0)	139 (28.2)	123 (30.6)	44 (29.7)	68 (30.1)	11 (39.3)
Antiplatelet drugs, n (%)	1457 (15.5)	1391 (15.5)	699 (15.7)	350 (14.9)	342 (15.7)	315 (16.3)	1005 (15.4)	71 (14.4)	66 (16.4)	25 (16.9)	39 (17.3)	2 (7.1)
Acetylsalicylic acid, n (%)	1419 (15.1)	1354 (15.1)	681 (15.3)	341 (14.5)	332 (15.3)	303 (15.7)	982 (15.0)	69 (14.0)	65 (16.2%)	25 (16.9)	38 (16.8)	2 (7.1)
Acetylsalicylic acid, combinations with proton pump inhibitors, n (%)	46 (0.5)	45 (0.5)	23 (0.5)	11 (0.5)	11 (0.5)	14 (0.7)	28 (0.4)	3 (0.6)	1 (0.3)		1 (0.4)	0
Antidepressants, n (%)	878 (9.4)	835 (9.3)	404 (9.1)	217 (9.2)	214 (9.8)	187 (9.7)	599 (9.2)	49 (9.9)	43 (10.7)	9 (6.1)	32 (14.2)	2 (7.1)
Anticancer drugs, n (%)	885 (9.4)	602 (6.7)	320 (7.2)	152 (6.5)	130 (6.0)	128 (6.6)	442 (6.8)	32 (6.5)	283 (70.4)	105 (71.0)	160 (70.8)	18 (64.3)
**Biochemical parameters**												
Hemoglobin, g/dL (SD)	12.1 (3.2)	12.1 (3.2)	12.2 (3.2)	11.97 (3.3)	11.92 (3.2)	12.1 (3.2)	12.1 (3.3)	12.4 (3.1)	11.8 (3.2)	11.7 (3.3)	11.74 (3.1)	12.2 (3.1)
HbA1c, % (SD)	7.4 (1.2)	7.4 (1.2)	7.5 (1.2)	7.2 (1.2)	7.58 (1.2)	7.5 (1.2)	7.4 (1.2)	7.5 (1.2)	7.2 (1.3)	7.3 (1.4)	7.2 (1.2)	7.1 (1.5)
Platelet count, x10^3/µL	276.9 (144.7)	277.4 (144.3)	278.1 (144.8)	262.8 (143.1)	291.62 (143.2)	277.1 (143.0)	278.1 (144.3)	269.0 (148.4)	265.7 (152.4)	258.8 (162.8)	266.4 (145.7)	296.1 (150.3)
eGFR, mL/min/1.73m^2 (SD)	94.7 (11.2)	94.7 (11.2)	94.84 (10.6)	94.8 (10.7)	94.2 (12.6)	94.9 (10.2)	94.6 (11.5)	95.1 (9.4)	94.3 (11.3)	94.6 (11.2)	94.0 (11.9)	95.9 (4.7)
Creatinine clearance, mL/min (SD)	50.8 (22.5)	50.8 (22.3)	49.44 (23.0)	52.6 (23.2)	51.7 (19.6)	51.1 (22.0)	50.8 (22.5)	50.8 (22.0)	50.4 (24.7)	49.3 (24.8)	51.7 (25.1)	46.5 (20.1)

[1]Dose as per label; [2]Dose higher than label; [3]Dose lower than label; [4]Consider: two tablets or capsules per day for apixaban and one per day for rivaroxaban and edoxaban; * within 180 days prior index. AF: atrial fibrillation; BMI: body mass index; DOACs: direct oral anticoagulants; eGFR: estimated glomerular filtration rate; FXai; Factor Xa inhibitors; FU: follow-up; MB: major bleeding; NSAIDs: non-steroidal anti-inflammatory drugs; SD: standard deviation; VTE: Venous thromboembolism

Supplementary Table S4. CHA_2D_2-VASC and HASBLED scores at index date and at 3 months from index date.

Time window	Overall FXai group N= 496								DOACs								Enoxaparin (active oncology patients only)							
	Overall	GIB	ICH	Trauma bleeding	Other	VTE	AF	Non-mechanical cardiac-valve replacement	Overall	GIB	ICH	Trauma bleeding	Other	VTE	AF	Non-mechanical cardiac-valve replacement	Overall	GIB	ICH	Trauma bleeding	Other	VTE	AF	Non-mechanical cardiac-valve replacement
**CHA_2D_2-VASc Score**																								
Mean (SD), at index date	3.4 (1.5)	3.4 (1.5)	3.4 (1.1)	2.9 (1.5)	3.5 (1.6)	3.3 (1.2)	3.4 (1.6)	3.7 (1.7)	3.4 (1.5)	3.4 (1.5)	3.5 (1.1)	3.0 (1.5)	3.6 (1.6)	3.3 (1.2)	3.4 (1.6)	3.9 (1.8)	3.0 (1.5)	3.0 (1.6)	3 (1.2)	2 (-)	2 (-)	3.1 (1.8)	2.9 (1.3)	3.0 (1.0)
Mean (SD), at 3 months from index date	4.8 (1.8)	4.8 (1.8)	5.0 (1.7)	4 .0 (1.7)	5.0 (1.6)	4.7 (1.6)	4.8 (1.8)	5.0 (1.9)	4.8 (1.8)	4.8 (1.8)	5.1 (1.7)	4.0 (1.7)	5.1 (1.6)	4.7 (1.5)	4.9 (1.8)	4.9 (1.9)	4.3 (1.7)	4.3 (1.9)	4.8 (1.5)	- (-)	3 (-)	4.3 (2.2)	4.3 (1.5)	6 (-)
**HAS-BLED Score**																								
Mean (SD), at index date	1.7 (1.1)	1.7 (1.1)	1.4 (1.1)	1.1 (1.0)	1.5 (0.9)	1.9 (1.2)	1.6 (1.1)	1.3 (1.0)	1.7 (1.1)	1.7 (1.1)	1.4 (1.2)	1.1 (1.0)	1.6 (0.9)	1.9 (1.2)	1.7 (1.1)	1.3 (1.0)	1.3 (1.2)	1.4 (1.4)	1.4 (0.5)	2 (-)	0 (-)	1.8 (1.6)	1.1 (1.0)	1.3 (0.6)
Mean (SD), at 3 months from index date	3.0 (1.4)	3.0 (1.4)	2.7 (1.3)	3.3 (1.2)	3.2 (1.3)	3.3 (1.4)	3.0 (1.3)	3.2 (1.0)	3.1 (1.3)	3.1 (1.3)	2.8 (1.3)	3.3 (1.2)	3.3 (1.3)	3.3 (1.3)	3.0 (1.45)	3.2 (1.1)	2.7 (1.7)	2.9 (1.9)	2.3 (1.0)	- (-)	2 (-)	3.5 (2.3)	2.2 (0.9)	3 (-)

AF: atrial fibrillation; DOACs: direct oral anticoagulants; FXai; Factor Xa inhibitors; GIB: gastrointestinal bleeding; ICH: intracranial hemorrhage; SD: standard deviation; VTE: Venous thromboembolism.

Supplementary Table S5. Major bleeding.

5a. Overall major bleeding event and incidence rates.

Time window	Outcome	OVERALL FXai GROUP (n=9374)				All DOACs (n=8972)				Dose as per label (n=7547)				Dose higher than label (n=685)				Dose lower than label (n=740)				Enoxaparin (n=402)			
		Outcomes (n)	Outcomes (%)	Event rate (95% C)	IR (95% CI)	Outcomes (n)	Outcomes (%)	Event rate (95% C)	IR (95% CI)	Outcomes (n)	Outcomes (%)	Event rate (95% C)	IR (95% CI)	Outcomes (n)	Outcomes (%)	Event rate (95% C)	IR (95% CI)	Outcomes (n)	Outcomes (%)	Event rate (95% C)	IR (95% CI)	Outcomes (n)	Outcomes (%)	Event rate (95% C)	IR (95% CI)
3 months	Major Bleeding	310	3.3	18.3 (17.6-19.1)	18.5 (17.7-19.3)	293	3.3	18.1 (17.3-18.9)	18.3 (17.5-19.1)	243	3.2	17.8 (16.9-18.6)	18.0 (17.1-18.8)	23	3.4	19.3 (16.4-22.3)	19.0 (16.0-21.9)	27	3.7	20.1 (17.3-23.0)	20.7 (17.7- 23.6)	17	4.2	24.6 (20.4-28.8)	25.1 (20.9-29.3)
	Critical site bleeding	21	0.2	1.3 (1.1-1.5)	1.3 (1.0- 1.5)	19	0.2	1.2 (0.9-1.4)	1.2 (1.0-1.4)	14	0.19	1.0 (0.8-1.2)	1.0 (0.8-1.3)	2	0.3	1.6 (0.7-2.6)	1.7 (0.7 – 2.6)	3	0.4	2.2 (1.2-3.3)	2.3 (1.2-3.4)	2	0.5	4.3 (2.4-6.3)	3.0 (1.3-4.6)
	Fatal	14	0.15	0.8 (0.6-1.0)	0.8 (0.7-1.0)	12	0.13	0.7 (0.6-0.9)	0.8 (0.6 – 0.9)	10	0.13	0.7 (0.5-0.9)	0.7 (0.6-0.9)	1	0.15	0.8 (0.1-1.5)	0.8 (0.1-1.5)	1	0.14	0.8 (0.1-1.4)	0.8 (0.1-1.4)	2	0.5	2.9 (1.3-4.5)	3.0 (1.3-4.6)
6 months	Major Bleeding	381	4.1	15.8 (15.1-16.6)	16.0 (15.3-16.7)	361	4.0	15.6 (14.9-16.4)	15.8 (15.0-16.5)	297	3.9	15.2 (14.3-16.0)	15.4 (14.5-16.2)	30	4.4	18.5 (15.6-21.4)	17.3 (14.5-20.2)	34	4.6	17.9 (15.1-20.6)	18.4 (15.6-21.2)	20	5.0	21.4 (17.4-25.4)	22.0 (18.0-26.1)
	Critical site bleeding	30	0.3	1.3 (1.0- 1.5)	1.3 (1.0-1.5)	27	0.3	1.2 (0.9-1.4)	1.2 (1.0-1.4)	21	0.3	1.1 (0.8-1.3)	1.1 (0.9- 1.3)	3	0.4	1.7 (0.7-2.6)	1.7 (0.8-2.7)	3	0.4	1.6 (0.7-2.5)	1.6 (0.7-2.5)	3	0.8	4.3 (2.3-6.3)	3.3 (1.6-5.1)
	Fatal	19	0.2	0.8 (0.6-1.0)	0.8 (0.6-1.0)	16	0.18	0.7 (0.5-0.9)	0.7 (0.5-0.9)	13	0.17	0.7 (0.5-0.8)	0.7 (0.5 – 0.9)	1	0.15	0.6 (0-1.1)	0.6 (0.01- 1.2)	2	0.3	1.1 (0.3-1.8)	1.1 (0.3-1.8)	3	0.8	3.2 (1.5-4.9)	3.3 (1.6-5.1)
12 months	Major Bleeding	433	4.6	14.4 (13.7- 15.1)	14.4 (13.7- 15.1)	410	4.6	14.2 (13.4-14.9)	14.2 (13.4- 14.9)	338	4.5	13.7 (12.9-14.5)	13.8 (13.0-14.6)	36	5.3	17.2 (14.4-20.0)	16.4 (13.6-19.1)	36	4.9	16.0 (13.4-18.7)	15.6 (12.9-18.2)	23	5.7	19.7 (15.8-23.6)	20.5 (16.5-24.4)
	Critical site bleeding	34	0.4	1.1 (0.9-1.4)	1.1 (0.9-1.3)	29	0.3	1.0 (0.8-1.2)	1 (0.8-1.2)	23	0.3	0.9 (0.7-1.2)	0.9 (0.7-1.2)	3	0.4	1.3 (0.4-2.1)	1.4 (0.5-2.2)	3	0.4	1.2 (0.4-2.0)	1.3 (0.5-2.1)	5	1.2	5.1 (3.0-7.3)	4.5 (2.4 – 6.5)
	Fatal	20	0.2	0.6 (0.5-0.8)	0.7 (0.5-0.8)	16	0.18	0.5 (0.4-0.7)	0.6 (0.4-0.7)	13	0.17	0.5 (0.4- 0.7)	0.5 (0.4-0.7)	1	0.15	0.4 (0-0.9)	0.5 (0– 0.9)	2	0.3	0.8 (0.2-1.5)	0.9 (0.2-1.5)	4	1.0	3.4 (1.6-5.2)	3.6 (1.8- 5.4)
2 years	Major Bleeding	462	4.9	12.2 (11.6-12.9)	12.4 (11.7- 13.0)	437	4.9	12.1 (11.4-12.7)	12.2 (11.5- 12.8)	359	4.8	11.5 (10.7-12.2)	11.9 (11.1- 12.6)	37	5.4	15.1 (12.4-17.8)	13.5 (11.0-16.1)	41	5.5	15.5 (12.9-18.1)	14.2 (11.7-16.7)	25	6.2	16.2 (12.6-19.8)	17.3 (13.6-21.0)
	Critical site bleeding	37	0.4	1.0 (0.8-1.2)	1.0 (0.8-1.2)	32	0.4	0.8 (0.7-1.0)	0.9 (0.7-1.1)	23	0.3	0.7 (0.5-0.9)	0.8 (0.6-1.0)	3	0.4	1.0 (0.2-1.7)	1.1 (0.3- 1.9)	6	0.8	1.9 (0.9-2.9)	2.1 (1.0-3.1)	5	1.2	3.9 (2.0-5.8)	3.5 (1.7-5.3)
	Fatal	21	0.2	0.6 (0.4-0.7)	0.6 (0.4-0.7)	17	0.19	0.5 (0.3-0.6)	0.5 (0.3-0.6)	13	0.17	0.4 (0.3-0.6)	0.4 (0.3-0.6)	1	0.15	0.7 (0.1-1.3)	0.4 (0-0.8)	3	0.4	1.0 (0.3-1.7)	1.0 (0.3-1.8)	4	1.0	2.6 (1.0-4.1)	2.8 (1.2-4.4)
3 years	Major Bleeding	479	5.1	11.2 (10.6-11.8)	11.5 (10.8- 12.1)	453	5.1	11.1 (10.4-11.7)	11.3 (10.7- 12.0)	372	4.9	10.5 (9.8-11.2)	11.0 (10.3-11.7)	39	5.7	13.7 (11.2-16.3)	12.8 (10.3-15.3)	42	5.7	14.0 (11.5-16.5)	12.9 (10.5-15.4)	26	6.5	14.4 (11.0-17.8)	16.0 (12.4-19.6)
	Critical site bleeding	40	0.4	0.9 (0.7- 1.1)	1.0 (0.8- 1.2)	34	0.4	0.8 (0.6-1.0)	0.9 (0.7- 1.0)	23	0.3	0.6 (0.5-0.8)	0.7 (0.5-0.9)	5	0.7	1.4 (0.5-2.3)	1.6 (0.7-2.6)	6	0.8	1.6 (0.7-2.5)	1.9 (0.9-2.8)	6	1.5	3.9 (2.0-5.8)	3.7 (1.9-5.6)
	Fatal	22	0.2	0.5 (0.4-0.7)	0.5 (0.4 – 0.7)	18	0.2	0.4 (0.3-0.6)	0.5 (0.3-0.6)	13	0.17	0.4 (0.2-0.5)	0.4 (0.3-0.5)	2	0.3	0.8 (0.2-1.5)	0.7 (0.1 – 1.3)	3	0.4	0.8 (0.2-1.5)	0.9 (0.2-1.6)	4	1.0	2.2 (0.8-3.7)	2.5 (1.0-4.0)
All	Major Bleeding	496	5.3	9.5 (8.9-10.1)	10.2 (9.6- 10.8)	470	5.2	9.4 (8.8-10.0)	10.1 (9.5-10.7)	386	5.1	9.1 (8.5-9.8)	9.9 (9.2-10.6)	39	5.7	10.3 (8.0-12.5)	10.8 (8.5-13.2)	45	6.1	11.1 (8.8-13.4)	11.3 (9.0-13.6)	26	6.5	11.4 (8.3-14.5)	13.1 (9.8-16.4)
	Critical site bleeding	41	0.4	0.7 (0.6-0.9)	0.8 (0.7-1.0)	35	0.4	0.6 (0.5-0.8)	0.8 (0.6-0.9)	23	0.3	0.5 (0.3- 0.7)	0.6 (0.4-0.8)	5	0.7	1.1 (0.3-1.8)	1.4 (0.5-2.3)	7	1.0	1.4 (0.6-2.2)	1.8 (0.8-2.7)	6	1.5	2.9 (1.2-4.5)	3.0 (1.4-4.7)
	Fatal	24	0.3	0.4 (0.3-0.6)	0.5 (0.4-0.6)	20	0.2	0.4 (0.3-0.5)	0.4 (0.3- 0.6)	15	0.2	0.3 (0.2-0.5)	0.4 (0.2-0.5)	2	0.3	0.6 (0.04 - 1.2)	0.6 (0 – 1.1)	3	0.4	0.6 (0.04-1.1)	0.8 (0.1-1.4)	4	1.0	1.6 (0.4-2.9)	2.0 (0.6-3.4)

5b. Overall major bleeding event and Incidence Rates according to FXai indication.

		Overall FXai GROUP (n=9374)				DOAC VTE (n=1932)				Enoxaparin VTE (n=148)				DOAC AF (n=6547)				Enoxaparin AF (n=226)				DOAC Non-mechanical cardiac-valve replacement (n=493)				Enoxaparin Non-mechanical cardiac-valve replacement (n=28)			
Time window	Outcome	Outcomes (n)	Outcomes (%)	Event rate (95% CI)	IR (95% CI)	Outcomes (n)	Outcomes (%)	Event rate (95% CI)	IR (95% CI)	Outcomes (n)	Outcomes (%)	Event rate (95% CI)	IR (95% CI)	Outcomes (n)	Outcomes (%)	Event rate (95% CI)	IR (95% CI)	Outcomes (n)	Outcomes (%)	Event rate (95% CI)	IR (95% CI)	Outcomes (n)	Outcomes (%)	Event rate (95% CI)	IR (95% CI)	Outcomes (n)	Outcomes (%)	Event rate (95% CI)	IR (95% CI)
3 months	Major Bleeding	310	3.3	18.3 (17.6-19.1)	18.5 (17.7-19.3)	47	2.4	13.8 (12.2-15.3)	13.7 (12.2-15.2)	7	4.7	28.0 (20.7-35.2)	28.6 (21.4-35.9)	232	3.5	19.5 (18.6-20.5)	19.8 (18.8-20.7)	9	4.0	22.6 (17.2-28.1)	23.1 (17.6-28.5)	14	2.8	15.4 (12.2-18.6)	15.7 (12.5-18.9)	1	3.6	23.6 (7.9-39.3)	23.6 (7.9-39.3)
	Critical site bleeding	21	0.2	1.3 (1.1-1.5)	1.3 (1.0- 1.5)	3	0.16	0.9 (0.5-1.3)	0.9 (0.5-1.3)	0	0	4.0 (0.8-7.1)	0	15	0.2	1.3 (1.0-1.5)	1.3 (1.0-1.6)	2	0.8	5.0 (2.2-7.9)	5.1 (2.3- 8.0)	1	0.2	1.1 (0.2-2.0)	1.1 (0.2-2.1)	0	0	0	0
	Fatal	14	0.15	0.8 (0.6-1.0)	0.8 (0.7-1.0)	0	0	0	0	1	0.7	4.0 (0.8-7.1)	4.1 (0.9-7.3)	9	0.14	0.8 (0.5-1.0)	0.8 (0.6-1.0)	1	0.4	2.5 (0.5-4.6)	2.6 (0.5-4.6)	3	0.6	3.3 (1.7-4.9)	3.4 (1.8-5.0)	0	0	0	0
6 months	Major Bleeding	381	4.1	15.8 (15.1-16.6)	16.0 (15.3-16.7)	62	3.2	12.9 (11.4-14.4)	12.8 (11.3-14.3)	7	4.7	20.8 (14.3-27.4)	21.4 (14.8-28.0)	283	4.3	16.6 (15.7-17.5)	16.9 (16.0-17.8)	11	4.9	20.3 (15.1-25.6)	21.0 (15.7-263)	16	3.3	12.9 (9.9-15.8)	12.3 (9.4-15.2)	2	7.1	35.5 (17.8-53.3)	35.5 (17.8-53.3)
	Critical site bleeding	30	0.3	1.3 (1.0- 1.5)	1.3 (1.0-1.5)	4	0.2	0.8 (0.4-1.2)	0.8 (0.4-1.2)	0	0	3.0 (0.2-5.7)	0	22	0.3	1.3 (1.0-1.5)	1.3 (1.0-1.6)	2	0.9	3.7 (1.2-6.2)	3.8 (1.3-6.3)	1	0.2	0.8 (0-1.5)	0.8 (0-1.5)	1	3.6	17.8 (3.6-31.9)	17.8 (3.6-31.9)
	Fatal	19	0.2	0.8 (0.6-1.0)	0.8 (0.6-1.0)	2	0.10	0.4 (0.1-0.7)	0.41 (0.1-0.7)	1	0.7	3.0 (0.2-5.7)	3.1 (0.3-5.8)	11	0.17	0.6 (0.5-0.8)	0.7 (0.5-0.9)	1	0.4	1.9 (0.1- 3.6)	1.9 (0.1 - 3.7)	3	0.6	2.3 (1.0-3.6)	2.3 (1.0-3.6)	1	3.6	17.8 (3.6-31.9)	17.8 (3.6-31.9)
12 month	Major Bleeding	433	4.6	14.4 (13.7- 15.1)	14.4 (13.7- 15.1)	71	3.7	11.5 (101 12.9)	11.7 (10.2-13.1)	8	5.4	18.6 (12.3-24.8)	18.9 (12.6-25.2)	321	4.9	15.1 (14.3-16.0)	15.1 (14.3-16.0)	12	5.3	17.9 (12.9-22.8)	18.8 (13.7-23.9)	18	3.7	11.2 (8.4-13.9)	10.8 (8.1-13.6)	3	10.7	46.5 (28.0-65.0)	46.5 (28.0- 65.0)
	Critical site bleeding	34	0.4	1.1 (0.9-1.4)	1.1 (0.9-1.3)	4	0.2	0.6 (0.3-1.0)	0.7 (0.3-1.0)	1	0.7	4.6 (1.3-8.0)	2.4 (0 - 4.8)	24	0.4	1.1 (0.9-1.4)	1.1 (0.9-1.4)	2	0.9	3.0 (0.8-5.2)	3.1 (0.9-5.4)	1	0.2	0.6 (0 - 1.3)	0.6 (0-1.3)	2	7.1	31.0 (13.9-48.1)	31.0 (13.9-48.1)
	Fatal	20	0.2	0.6 (0.5-0.8)	0.7 (0.5-0.8)	2	0.10	0.3 (0.1-0.6)	0.3 (0.1-0.6)	2	1.4	4.6 (1.3-8.0)	4.7 (1.3 - 8.1)	11	0.17	0.5 (0.3-0.7)	0.5 (0.4-0.7)	1	0.4	1.5 (0-3.1)	1.6 (0-3.2)	3	0.6	1.8 (0.6 - 2.9)	1.8 (0.6 – 3.0)	1	3.6	15.5 (2.1-28.9)	15.5 (2.1-28.9)
2 years	Major Bleeding	462	4.9	12.2 (11.6-12.9)	12.4 (11.7- 13.0)	75	3.9	9.9 (8.5-11.2)	10.0 (8.6-11.3)	10	6.8	17.3 (11.2-23.4)	17.7 (11.6-23.9)	342	5.2	12.9 (12.1-13.7)	13.0 (12.2-13.8)	12	5.3	13.4 (9.0-17.8)	14.8 (10.2-19.4)	20	4.1	9.5 (6.9-12.0)	9.5 (6.9-12.1)	3	10.7	42.7 (24.4-61.0)	42.7 (24.4-61.0)
	Critical site bleeding	37	0.4	1.0 (0.8-1.2)	1.0 (0.8-1.2)	5	0.3	0.6 (0.3-1.0)	0.7 (0.3-1.0)	1	0.7	3.5 (0.5-6.4)	1.8 (0-3.9)	26	0.4	0.9 (0.7-1.2)	1.0 (0.8- 1.2)	2	0.9	2.2 (0.3-4.2)	2.5 (0.5-4.5)	1	0.2	0.5 (0 - 1.0)	0.5 (0 - 1.1)	2	7.1	28.5 (11.7-45.2)	28.5 (11.7-45.2)
	Fatal	21	0.2	0.6 (0.4-0.7)	0.6 (0.4-0.7)	2	0.10	0.2 (0.02-0.5)	0.3 (0.04-0.5)	2	1.4	3.4 (0.5-6.4)	3.5 (0.6-6.5)	12	0.18	0.5 (0.3-0.7)	0.5 (0.3-0.6)	1	0.4	1.1 (0-2.5)	1.2 (0-2.7)	3	0.6	1.4 (0.3-2.4)	1.4 (0.4-2.5)	1	3.6	14.2 (1.3-27.2)	14.2 (1.3-27.2)
3 years	Major Bleeding	479	5.1	11.2 (10.6-11.8)	11.5 (10.8- 12.1)	76	3.9	8.5 (7.3-9.8)	9.0 (7.7-10.3)	10	6.8	14.7 (9.0-20.4)	15.7 (9.8-21.5)	357	5.5	12.0 (11.2-12.8)	12.2 (11.4-13.0)	13	5.8	12.3 (8.0-16.6)	14.2 (9.7-18.8)	20	4.1	8.1 (5.7-10.5)	8.5 (6.1-11.0)	3	10.7	42.7 (24.4-61.0)	42.7 (24.4-61.0)
	Critical site bleeding	40	0.4	0.9 (0.7- 1.1)	1.0 (0.8- 1.2)	5	0.3	0.5 (0.2-0.8)	0.6 (0.3-0.9)	1	0.7	2.9 (0.2-5.7)	1.6 (0-3.6)	28	0.4	0.9 (0.7-1.1)	1.0 (0.7-1.2)	3	1.3	2.8 (0.7-5.0)	3.3 (1.0-5.6)	1	0.2	0.4 (0 - 0.9)	0.4 (0-1.0)	2	7.1	28.5 (11.7-45.2)	28.5 (11.7-45.2)
	Fatal	22	0.2	0.5 (0.4-0.7)	0.5 (0.4 - 0.7)	2	0.10	0.2 (0.01-0.4)	0.2 (0.02-0.5)	2	1.4	2.9 (0.2-5.7)	3.1 (0.3-5.9)	13	0.2	0.5 (0.3-0.6)	0.4 (0.3-0.6)	1	0.4	1.0 (0-2.2)	1.1 (0-2.5)	3	0.6	1.2 (0.2-2.1)	1.3 (0.3-2.3)	1	3.6	14.2 (1.3-27.2)	14.2 (1.3-27.2)
All	Major Bleeding	496	5.3	9.5 (8.9-10.1)	10.2 (9.6- 10.8)	83	4.3	7.4 (6.2-8.5)	8.5 (7.2-9.7)	10	6.8	9.7 (4.9-14.5)	12.8 (7.4-18.2)	367	5.6	10.2 (9.5-10.9)	10.8 (10.0-11.5)	13	5.8	11.0 (7.0-15.1)	11.5 (7.3-15.6)	20	4.1	6.5 (4.4-8.7)	7.4 (5.1-9.7)	3	10.7	42.7 (24.4-61.0)	42.7 (24.4-61.0)
	Critical site bleeding	41	0.4	0.7 (0.6-0.9)	0.8 (0.7-1.0)	5	0.3	0.4 (0.1-0.7)	0.5 (0.2-0.8)	1	0.7	1.9 (0-4.2)	1.3 (0-3.1)	29	0.4	0.7 (0.5-0.9)	0.9 (0.6- 1.1)	3	1.3	2.2 (0.3-4.1)	2.6 (0.6-4.7)	1	0.2	0.3 (0 - 0.8)	0.4 (0-0.9)	2	7.1	28.5 (11.7-45.2)	28.5 (11.7-45.2)
	Fatal	24	0.3	0.4 (0.3-0.6)	0.5 (0.4-0.6)	3	0.16	0.2 (0.02-0.5)	0.3 (0.06-056)	2	1.4	1.9 (0-4.2)	2.6 (0.02 - 5.1)	14	0.2	0.4 (0.2-0.5)	0.4 (0.3-0.6)	1	0.4	0.7 (0-1.9)	0.9 (0-2.1)	3	0.6	0.9 (0.1-1.8)	1.1 (0.2-2.0)	1	3.6%	14.2 (1.3-27.32)	14.2 (1.3-27.2)

5c. Overall major bleeding event and incidence rates according to the type of direct FXai.

Time window	Outcome	All DOACs (n=8972)				Apixaban (n=4441)				Rivaroxaban (n=2355)				Edoxaban (n=2176)			
		Outcomes (n)	Outcomes (%)	Event rate (95% C)	IR (95% CI)	Outcomes (n)	Outcomes (%)	Event rate (95% C)	IR (95% CI)	Outcomes (n)	Outcomes (%)	Event rate (95% C)	IR (95% CI)	Outcomes (n)	Outcomes (%)	Event rate (95% C)	IR (95% CI)
3 months	Major Bleeding	293	3.3	18.1 (17.3-18.9)	18.3 (17.5-19.1)	137	3.1	16.5 (15.4-17.6)	16.7 (15.6-17.8)	80	3.4	19.4 (17.8-21.0)	19.4 (17.8-21.0)	76	3.5	20.1 (18.4-21.8)	20.6 (18.9-22.3)
	Critical site bleeding	19	0.2	1.2 (0.9-1.4)	1.2 (1.0-1.4)	9	0.2	1.1 (0.8-1.4)	1.1 (0.8-1.4)	4	0.2	1.0 (0.6-1.3)	1.0 (0.6-1.4)	6	0.3	1.6 (1.1-2.1)	1.6 (1.1-2.2)
	Fatal	12	0.13	0.7 (0.6-0.9)	0.8 (0.6 – 0.9)	8	0.2	1.0 (0.7-1.3)	1.0 (0.7-1.3)	3	0.1	0.7 (0.4-1.1)	0.7 (0.4-1.1)	1	0.1	0.3 (0.1-0.5)	0.3 (0.1-0.5)
6 months	Major Bleeding	361	4.0	15.6 (14.9-16.4)	15.8 (15.0-16.5)	175	3.9	14.4 (13.4-15.4)	14.4 (134.4-15.5)	94	4.0	16.1 (14.6-17.5)	16.2 (14.7-17.7)	92	4.2	18.1 (16.5-19.7)	18.5 (16.9-20.11)
	Critical site bleeding	27	0.3	1.2 (0.9-1.4)	1.2 (1.0-1.4)	14	0.3	1.1 (0.8-1.4)	1.2 (0.8-1.5)	7	0.3	1.2 (0.7-1.6)	1.2 (0.8-1.7)	6	0.3	1.2 (0.7-1.6)	1.2 (0.8-1.7)
	Fatal	16	0.18	0.7 (0.5-0.9)	0.7 (0.5-0.9)	12	0.3	1.0 (0.7-1.3)	1.0 (0.7-1.3)	3	0.1	0.5 (0.2-0.8)	0.5 (0.2-0.8)	1	0.1	0.2 (0.01-0.4)	0.2 (0.01-0.4)
12 months	Major Bleeding	410	4.6	14.2 (13.4-14.9)	14.2 (13.4- 14.9)	205	4.6	13.2 (12.2-14.2)	13.2 (12.2-14.2)	101	4.3	13.4 (12.5-15.3)	13.8 (12.4-15.2)	104	4.8	16.8 (15.2-18.4)	17.0 (15.5-18.6)
	Critical site bleeding	29	0.3	1.0 (0.8-1.2)	1 (0.8-1.2)	16	0.4	1.0 (0.7-1.3)	1.0 (0.7-1.3)	8	0.3	1.0 (0.6-1.5)	1.0 (0.6-1.4)	6	0.32	0.9 (0.5-1.3)	1.0 (0.6-1.4)
	Fatal	16	0.18	0.5 (0.4-0.7)	0.6 (0.4-0.7)	12	0.3	0.7 (0.5-1.0)	0.8 (0.5-1.0)	3	0.1	0.4 (0.1-0.6)	0.4 (0.2-0.7)	1	0.05	0.2 (0 -0.3)	0.2 (0-0.3)
2 years	Major Bleeding	437	4.9	12.1 (11.4-12.7)	12.2 (11.5- 12.8)	220	5.0	11.3 (10.3-12.2)	11.4 (10.5-12.3)	109	4.6	12.2 (10.9-13.6)	11.9 (10.6-13.2)	108	5.0	13.9 (12.4-15.3)	14.5 (13.0-15.9)
	Critical site bleeding	32	0.4	0.8 (0.7-1.0)	0.9 (0.7-1.1)	18	0.4	0.9 (0.6-1.1)	0.9 (0.7-1.2)	8	0.3	0.8 (0.4-1.2)	0.8 (0.4-1.1)	7	0.3	0.8 (0.5-1.2)	0.9 (0.5-1.4)
	Fatal	17	0.19	0.5 (0.3-0.6)	0.5 (0.3-0.6)	14	0.3	0.7 (0.4-0.9)	0.7 (0.4-0.9)	4	0.2	0.4 (0.2-0.7)	0.3 (0.1-0.6)	1	0.05	0.1 (0-0.3)	0.1 (0-0.3)
3 years	Major Bleeding	453	5.1	11.1 (10.4-11.7)	11.3 (10.7- 12.0)	228	5.1	10.3 (9.4-11.1)	10.6 (9.7-11.5)	112	4.8	11.2 (9.9-12.4)	10.9 (9.6-12.1)	113	5.2	13.0 (11.6-14.4)	13.7 (12.2-15.1)
	Critical site bleeding	34	0.4	0.8 (0.6-1.0)	0.9 (0.7- 1.0)	19	0.4	0.8 (0.5-1.1)	0.9 (0.6-1.2)	9	0.4	0.8 (0.4-1.1)	0.8 (0.4-1.1)	7	0.3	0.7 (0.4-1.1)	0.9 (0.5-1.2)
	Fatal	18	0.2	0.4 (0.3-0.6)	0.5 (0.3-0.6)	14	0.3	0.6 (0.4-0.8)	0.6 (0.4-0.8)	5	0.2	0.4 (0.2- 0.7)	0.4 (0.1-0.6)	1	0.05	0.1 (0-0.2)	0.1 (0-0.3)
All	Major Bleeding	470	5.2	9.4 (8.8-10.0)	10.1 (9.5-10.7)	237	5.3	8.7 (7.9-9.5)	9.6 (8.7-10.4)	114	4.8	9 (7.8-10.2)	8.9 (7.7-10.0)	119	5.5	11.6 (10.3-13.0)	13.2 (11.8-14.6)
	Critical site bleeding	35	0.4	0.6 (0.5-0.8)	0.8 (0.6-0.9)	19	0.4	0.6 (0.4-0.9)	0.8 (0.5-1.0)	9	0.4	0.6 (0.3-0.9)	0.6 (0.3-0.9)	8	0.4	0.7 (0.3-1.0)	0.9 (0.5-1.3)
	Fatal	20	0.2	0.4 (0.3-0.5)	0.4 (0.3- 0.6)	16	0.4	0.5 (0.3-0.7)	0.6 (0.4-0.8)	5	0.2	0.3 (0.1-056)	0.3 (0.1-0.5)	1	0.05	0.1 (0-0.2)	0.1 (0-0.3)

AF: atrial fibrillation; DOACs: direct oral anticoagulants; Fxai; Factor Xa inhibitors; IR (95% CI): Incidence rate (95% confidence interval); VTE: venous thromboembolism. Time window: Cumulative bleedings from index (day of the first recording with DOAC or enoxaparin in active oncology patients) to the specific time window; Outcome: first bleeding; Event rate (events per 100 person-years) was defined as as the total number of bleeding events, including recurrent events divided by the total person-time of follow-up. Incidence rate (IR, events per 100 person-years) was defined as the total number of incident bleeding events divided by the total person-time at risk; Enoxaparin group: active oncology patients only; N: number of patients in the total group or subgroup or with the event.

Supplementary Table S6. Major gastrointestinal bleeding.

6a. Major gastrointestinal bleeding event and incidence rates.

Time window	Outcome	All FXai (n=9374)				All DOACS (n=8972)				Enoxaparin (n=402)			
		Outcomes (n)	Outcomes (%)	Event rate (95% C)	IR (95% CI)	Outcomes (n)	Outcomes (%)	Event rate (95% C)	IR (95% CI)	Outcomes (n)	Outcomes (%)	Event rate (95% CI)	IR (95% CI)
3 months	Major GIB	275	2.9	16.3 (15.5-17.0)	16.4 (15.7-17.2)	261	2.9	16.1 (15.4-16.9)	16.3 (15.5-17.0)	14	3.5	20.3 (16.3-24.2)	20.7 (19.8-21.5)
	Fatal	6	0.06	0.4 (0.2-0.5)	0.4 (0.2-0.5)	5	0.06	0.3 (0.2-0.4)	0.3 (0.2-0.4)	1	0.2	1.5 (0.3-2.6)	1.5 (1.2-1.7)
6 months	Major GIB	334	3.6	13.9 (13.2-14.6)	14.0 (13.3-14.7)	318	3.5	13.8 (13.1-14.5)	13.9 (13.2-14.6)	16	4	17.1 (13.5-20.8)	17.6 (16.8-18.4)
	Fatal	8	0.09	0.3 (0.2-0.5)	0.3 (0.2-0.5)	7	0.08	0.3 (0.2-0.4)	0.3 (0.2-0.4)	1	0.2	1.1 (0.1-2.1)	1.1 (0.9-1.3)
12 months	Major GIB	382	4.1	12.7 (12.0-13.4)	12.7 (12.0-13.4)	365	4.1	12.6 (11.9-13.3)	12.6 (11.9-13.3)	17	4.2	14.6 (11.1-18.0)	15.1 (14.4-15.9)
	Fatal	8	0.09	0.3 (0.2 - 0.4)	0.3 (0.2-0.4)	7	0.08	0.2 (0.1-0.3)	0.2 (0.1-0.3)	1	0.2	0.9 (0-1.8)	0.9 (0.7-1.1)
2 years	Major GIB	407	4.3	10.8 (10.2 11.5)	10.9 (10.3-11.5)	389	4.3	10.8 (10.2-11.5)	10.8 (10.2-11.5)	18	4.5	11.7 (8.5-14.8)	12.5 (11.8-13.1)
	Fatal	8	0.08	0.3 (0.2-0.4)	0.2 (0.1-0.3)	7	0.08	0.2 (0.1-0.3)	0.2 (0.1-0.3)	1	0.2	0.7 (0-1.4)	0.7 (0.5-0.9)
3 years	Major GIB	420	4.5	9.8 (9.2-10.4)	10.1 (9.5-10.7)	402	4.5	9.8 (9.2-10.5)	10.0 (9.4-10.7)	18	4.5	10.0 (7.0-12.9)	11.1 (10.5-11.8)
	Fatal	8	0.09	0.2 (0.1-0.3)	0.2 (0.1-0.3)	7	0.08	0.2 (0.1-0.3)	0.2 (0.1-0.3)	1	0.2	0.6 (0-1.3)	0.6 (0.5-0.8)
All	Major GIB	436	4.7	8.4 (7.9-9.0)	9.0 (8.4-9.5)	418	4.7	8.4 (7.9-9.0)	9.0 (8.4-9.6)	18	4.5	8.1 (5.5-10.8)	9.1 (8.5-9.7)
	Fatal	10	0.1	0.2 (0.1-0.3)	0.2 (0.1-0.3)	9	0.1	0.2 (0.1-0.3)	0.2 (0.1 - 0.3)	1	0.2	0.4 (0-1.0)	0.5 (0.4-0.7)

6b. Major gastrointestinal bleeding event and incidence rates according to the type of gastrointestinal bleeding.

All FXai (n=9374)

Time window	Outcome	Upper				Lower				Unspecified			
		Outcomes (n)	Outcomes (%)	Event rate (95% C)	IR (95% CI)	Outcomes (n)	Outcomes (%)	Event rate (95% C)	IR (95% CI)	Outcomes (n)	Outcomes (%)	Event rate (95% C)	IR (95% CI)
3 months	Major GIB	167	1.8	9.8 (9.2-10.4)	10.0 (9.4- 10.6)	74	0.8	4.4 (4.0-4.8)	4.4 (4.0-4.8)	34	0.4	2.1 (1.8 2.4)	2.0 (1.7-2.3)
	Fatal	3	0.03	0.2 (0.1-0.3)	0.2 (0.1-0.3)	2	0.02	0.1 (0.05-0.2)	0.1 (0.05-0.2)	1	0.01	0.1 (0.01-0.1)	0.1 (0.01-0.1)
6 months	Major GIB	202	2.2	8.3 (7.8-8.9)	8.5 (7.9-9.0)	86	0.9	3.6 (3.3-4.0)	3.6 (3.2-4.0)	46	0.5	2.0 (1.7-2.2)	1.9 (1.7-2.2)
	Fatal	4	0.04	0.2 (0.1-0.2)	0.2 (0.1-0.3)	2	0.02	0.1 (0.02-0.1)	0.1 (0.02-0.1)	2	0.02	0.1 (0.02-0.1)	0.1 (0.02-0.1)
12 months	Major GIB	235	2.5	7.7 (7.1-8.2)	7.8 (7.3-8.4)	97	1.0	3.4 (3.0-3.7)	3.2 (2.9-3.6)	50	0.5	1.7 (1.4-1.9)	1.7 (1.4-1.9)
	Fatal	4	0.04	0.1 (0.1-0.2)	0.1 (0.1-0.2)	2	0.02	0.1 (0.01-0.1)	0.1 (0.02-0.1)	2	0.02	0.1 (0.01-0.1)	0.1 (0.02-0.1)
2 years	Major GIB	249	2.7	6.5 (6.0-7.0)	6.7 (6.2-7.2)	104	1.1	2.8 (2.5-3.2)	2.8 (2.5-3.1)	54	0.6	1.5 (1.3-1.8)	1.4 (1.2-1.7)
	Fatal	4	0.04	0.2 (0.1- 0.2)	0.1 (0.04-0.2)	2	0.02	0.05 (0-0.1)	0.05 (0-0.1)	2	0.02	0.05 (0-0.1)	0.05 (0-0.1)
3 years	Major GIB	257	2.7	6.0 (5.5-6.4)	6.2 (5.7-6.7)	108	1.2	2.6 (2.3-2.9)	2.6 (2.3-2.9)	55	0.6	1.3 (1.1-1.5)	1.3 (1.1-1.6)
	Fatal	4	0.04	0.1 (0.1-0.2)	0.1 (0.04-0.2)	2	0.02	0.04 (0-0.1)	0.05 (0-0.1)	2	0.02	0.04 (0-0.1)	0.05 (0-0.1)
All	Major GIB	264	2.8	5.0 (4.6-5.4)	5.4 (5.0 -5.9)	114	1.2	2.3 (2.0-2.6)	2.3 (2.0-2.7)	59	0.6	1.2 (0.9-1.4)	1.2 (1.0-1.4)
	Fatal	6	0.06	0.1 (0.1-0.2)	0.1 (0.05-0.2)	2	0.02	0.03 (0-0.1)	0.04 (0-0.1)	2	0.02	0.03 (0-0.1)	0.04 (0-0.1)

All DOACS (n=8972)

Time window	Outcome	Upper				Lower				Unspecified			
		Outcomes (n)	Outcomes (%)	Event rate (95% C)	IR (95% CI)	Outcomes (n)	Outcomes (%)	Event rate (95% C)	IR (95% CI)	Outcomes (n)	Outcomes (%)	Event rate (95% C)	IR (95% CI)
3 months	Major GIB	156	1.7	9.5 (8.9-10.1)	9.7 (9.1-10.3)	73	0.8	4.5 (4.1-5.0)	4.6 (4.1-5.0)	32	0.4	2.1 (1.8-2.4)	2.0 (1.7-2.3)
	Fatal	3	0.03	0.2 (0.1-0.3)	0.2 (0.1-0.3)	2	0.02	0.1 (0.05-0.2)	0.1 (0.05-0.2)	0	0	0	0
6 months	Major GIB	189	2.1	8.1 (7.6-8.7)	8.3 (7.7-8.8)	85	1.0	3.7 (3.4-4.1)	3.7 (3.3-4.1)	44	0.5	2.0 (1.7-2.2)	1.9 (1.6-2.2)
	Fatal	4	0.04	0.2 (0.1-0.3)	0.2 (0.1-0.3)	2	0.02	0.1 (0.02-0.1)	0.1 (0.03-0.2)	1	0.01	0.04 (0-0.1)	0.04 (0-0.1)
12 months	Major GIB	222	2.5	7.5 (7.0-8.1)	7.7 (7.1-8.2)	95	1.1	3.4 (3.1-3.8)	3.3 (2.9-3.7)	48	0.5	1.7 (1.4-2.0)	1.7 (1.4-1.9)
	Fatal	4	0.04	0.1 (0.1-0.2)	0.1 (0.1-0.2)	2	0.02	0.1 (0.02-0.1)	0.1 (0.02-0.1)	1	0.01	0.03 (0-0.1)	0.03 (0-0.07)
2 years	Major GIB	236	2.6	6.4 (5.9-6.9)	6.6 (6.1-7.1)	102	1.1	2.9 (2.6-3.3)	2.8 (2.5-3.2)	51	0.6	1.5 (1.2-1.7)	1.4 (1.2-1.7)
	Fatal	4	0.04	0.2 (0.1-0.2)	0.1 (0.04-0.2)	2	0.02	0.05 (0-0.1)	0.1 (0.01-0.1)	1	0.01	0.03 (0-1.0)	0.03 (0-0.07)
3 years	Major GIB	244	2.7	5.9 (5.4-6.4)	6.1 (5.6-6.6)	106	1.2	2.7 (2.3-3.0)	2.7 (2.3-3.0)	52	0.6	1.3 (1.1-1.5)	1.3 (1.1-1.5)
	Fatal	4	0.04	0.1 (0.1-0.2)	0.1 (0.03-0.2)	2	0.02	0.04 (0-0.1)	0.05 (0-0.1)	1	0.01	0.02 (0-0.05)	0.02 (0-0.05)
All	Major GIB	251	2.8	4.9 (4.5-5.4)	5.4 (4.9-5.8)	112	1.3	2.4 (2.1-2.7)	2.4 (2.1-2.7)	56	0.6	1.1 (0.9-1.4)	1.2 (1.0-1.4)
	Fatal	6	0.1	0.1 (0.1-0.2)	0.1 (0.1-0.2)	2	0.02	0.03 (0-0.1)	0.04 (0-0.1)	1	0.01	0.02 (0-0.05)	0.02 (0-0.05)

Enoxaparin (n=402)

Time window	Outcome	Upper				Lower				Unspecified			
		Outcomes (n)	Outcomes (%)	Event rate (95% C)	IR (95% CI)	Outcomes (n)	Outcomes (%)	Event rate (95% C)	IR (95% CI)	Outcomes (n)	Outcomes (%)	Event rate (95% C)	IR (95% CI)
3 months	Major GIB	11	2.7	15.9 (12.3-19.5)	16.2 (15.5-17.0)	1	0.3	1.5 (0.3-2.6)	1.5 (1.2-1.7)	2	0.5	2.9 (1.3-4.5)	3.0 (2.6-3.3)
	Fatal	0	0	0	0	0	0	0	0	1	0.3	1.5 (0.3-2.6)	1.5 (1.2-1.7)
6 months	Major GIB	13	3.2	13.9 (10.6-17.3)	14.3 (13.6-15.0)	1	0.3	1.1 (0.1-2.1)	1.1 (0.9-1.3)	2	0.5	2.1 (0.7-3.6)	2.2 (1.9-2.5)
	Fatal	0	0	0	0	0	0	0	0	1	0.3	1.1 (0.1-2.1)	1.1 (0.9-1.3)
12 months	Major GIB	13	3.2	11.1 (8.1-14.2)	11.6 (10.9-12.2)	2	0.5	1.7 (0.4-3.0)	1.8 (1.5-2.1)	2	0.5	1.7 (0.4-3.0)	1.8 (1.5-2.1)
	Fatal	0	0	0	0	0	0	0	0	1	0.3	0.9 (0-1.8)	0.9 (0.7-1.1)
2 years	Major GIB	13	3.2	8.4 (5.7-11.1)	9.0 (8.4-9.6)	2	0.5	1.3 (0.2-2.4)	1.4 (1.1-1.6)	3	0.8	1.9 (0.6-3.3)	2.1 (1.7-2.4)
	Fatal	0	0	0	0	0	0	0	0	1	0.3	0.7 (0-1.4)	0.7 (0.5-0.9)
3 years	Major GIB	13	3.2	7.2 (4.7-9.7)	8.0 (7.5-8.6)	2	0.5	1.1 (0.1-2.1)	1.2 (1.0-1.5)	3	0.8	1.7 (0.4-2.9)	1.9 (1.6-2.1)
	Fatal	0	0	0	0	0	0	0	0	1	0.3	0.6 (0-1.3)	0.6 (0.5-0.8)
All	Major GIB	13	3.2	6.1 (3.8-8.4)	6.5 (6.0-7.1)	2	0.5	0.8 (0-1.7)	1.0 (0.8-1.2)	3	0.8	1.2 (0.2-2.3)	1.5 (1.3-1.8)
	Fatal	0	0	0	0	0	0	0	0	1	0.3	0.4 (0-1.0)	0.5 (0.4-0.7)

DOACs: direct oral anticoagulants; FXai; Factor Xa inhibitors; GIB: gastrointestinal bleeding; IR (95% CI): Incidence rate (95% confidence interval). Time window: Cumulative bleedings from index (day of the first recording with DOAC or enoxaparin in active oncology patients) to the specific time window; Outcome: first bleeding; Event rate (events per 100 person-years) was defined as as the total number of bleeding events, including recurrent events divided by the total person-time of follow-up. Incidence rate (IR, events per 100 person-years) was defined as the total number of incident bleeding events divided by the total person-time at risk; Enoxaparin group: active oncology patients only; N: number of patients in the total group or subgroup or with the event.

Supplementary Table S7. Intracranial bleeding.

7a. Intracranial bleeding event and incidence rates.

Time window	Outcome	All FXai (n=9374)				All DOACS (n=8972)				Enoxaparin (n=402)			
		Outcomes (n)	Outcomes (%)	Event rate (95% C)	IR (95% CI)	Outcomes (n)	Outcomes (%)	Event rate (95% C)	IR (95% CI)	Outcomes (n)	Outcomes (%)	Event rate (95% C)	IR (95% CI)
3 months	ICH	20	0.2	1.2 (1.0-1.4)	1.2 (1.0-1.4)	17	0.2	1.0 (0.8-1.3)	1.1 (0.9-1.3)	3	0.8	4.3 (2.4-6.3)	4.4 (2.4-6.4)
	Fatal	7	0.07	0.4 (0.3-0.5)	0.4 (0.3-0.6)	6	0.07	0.37 (0.2-0.5)	0.4 (0.2-0.5)	1	0.3	1.5 (0.3-2.6)	1.5 (0.3-2.7)
6 months	ICH	28	0.3	1.1 (0.9-1.4)	1.2 (1.0-1.4)	24	0.3	1.0 (0.8-1.2)	1.1 (0.8-1.3)	4	1.0	4.3 (2.3-6.3)	4.4 (2.4-6.4)
	Fatal	9	0.1	0.4 (0.3-0.5)	0.4 (0.3-0.5)	7	0.08	0.3 (0.2-0.4)	0.3 (0.2-0.4)	2	0.5	2.1 (0.7-3.6)	2.2 (0.8-3.6)
12 months	ICH	32	0.3	1.1 (0.8-1.3)	1.1 (0.9-1.3)	26	0.3	0.9 (0.7-1.1)	0.9 (0.7-1.1)	6	1.5	5.1 (3.0-7.3)	5.3 (3.1-7.5)
	Fatal	10	0.1	0.3 (0.2-0.4)	0.3 (0.2-0.5)	7	0.08	0.2 (0.1-0.3)	0.2 (0.1-0.3)	3	0.8	2.6 (1.0-4.1)	2.7 (1.1-4.3)
2 years	ICH	35	0.4	0.9 (0.7-1.1)	0.9 (0.7-1.1)	29	0.3	0.8 (0.6-0.9)	0.8 (0.6-1.0)	6	1.5	3.9 (2.0-5.8)	4.2 (2.2-6.1)
	Fatal	11	0.1	0.3 (0.2-0.4)	0.3 (0.2-0.4)	8	0.09	0.2 (0.1-0.3)	0.2 (0.1-0.3)	3	0.8	1.9 (0.6-3.3)	2.1 (0.7-3.5)
3 years	ICH	38	0.4	0.8 (0.6-1.0)	0.9 (0.7-1.1)	31	0.4	0.7 (0.5-0.9)	0.8 (0.6-1.0)	7	1.7	3.9 (2.0-5.8)	4.3 (2.3-6.3)
	Fatal	12	0.1	0.3 (0.2-0.4)	0.3 (0.2-0.4)	9	0.1	0.2 (0.1-0.3)	0.2 (0.1-0.3)	3	0.8	1.7 (0.4-2.9)	1.9 (0.5-3.2)
All	ICH	39	0.4	0.7 (0.5-0.8)	0.8 (0.6-1.0)	32	0.4	0.6 (0.4-0.7)	0.7 (0.5-0.9)	7	1.7	2.9 (1.2-4.5)	3.5 (1.7-5.3)
	Fatal	12	0.1	0.2 (0.1-0.3)	0.3 (0.2-0.4)	9	0.1	0.2 (0.1-0.2)	0.2 (0.1-0.3)	3	0.8	1.2 (0.2-2.3)	1.5 (0.3-2.7)

7b. Intracranial bleeding event and incidence rates according to the type of intracranial bleeding.

All FXai (n=9374)

Time window	Outcome	Intracerebral/intraventricular hemorrhage				Subarachnoid hemorrhage				Subdural, epidural and extradural hemorrhages			
		Outcomes (n)	Outcomes (%)	Event rate (95% C)	IR (95% CI)	Outcomes (n)	Outcomes (%)	Event rate (95% C)	IR (95% CI)	Outcomes (n)	Outcomes (%)	Event rate (95% C)	IR (95% CI)
3 months	ICH	10	0.1	0.6 (0.4-0.8)	0.6 (0.4-0.8)	8	0.1	0.5 (0.3-0.6)	0.5 (0.3-0.6)	2	0.02	0.1 (0.05-0.2)	0.1 (0.05-0.2)
	Fatal	3	0.03	0.2 (0.1-0.3)	0.2 (0.1-0.3)	2	0.02	0.1 (0.05-0.2)	0.1 (0.05 - 0.2)	2	0.02	0.1 (0.05-0.2)	0.1 (0.05-0.2)
6 months	ICH	14	0.2	0.6 (0.4-0.7)	0.6 (0.4-0.8)	11	0.1	0.5 (0.3-0.6)	0.5 (0.3-0.6)	3	0.03	0.1 (0.05-0.2)	0.1 (0.06-0.2)
	Fatal	3	0.03	0.1 (0.05-0.2)	0.1 (0.06-0.2)	4	0.04	0.2 (0.1-0.2)	0.2 (0.1-0.3)	2	0.02	0.1 (0.02-0.1)	0.1 (0.02-0.1)
12 months	ICH	17	0.2	0.5 (0.4-0.7)	0.5 (0.4-0.7)	13	0.1	0.4 (0.3-0.5)	0.4 (0.3-0.6)	3	0.03	0.1 (0.04-0.2)	0.1 (0.04-0.2)
	Fatal	3	0.03	0.1 (0.04-0.2)	0.1 (0.04-0.2)	5	0.05	0.2 (0.1-0.2)	0.2 (0.1-0.3)	2	0.02	0.06 (0.01-0.1)	0.1 (0.02-0.1)
2 years	ICH	17	0.2	0.4 (0.3-0.6)	0.4 (0.3-0.6)	16	0.2	0.4 (0.3-0.5)	0.4 (0.3-0.6)	3	0.03	0.1 (0.02-0.1)	0.1 (0.02-0.1)
	Fatal	3	0.03	0.1 (0.02-0.1)	0.1 (0.02-0.1)	6	0.06	0.2 0.1-0.2)	0.2 (0.1-0.2)	2	0.02	0.05 (0-0.1)	0.05 (0-0.1)
3 years	ICH	20	0.2	0.4 (0.3-0.6)	0.5 (0.3-0.6)	16	0.2	0.3 (0.2-0.5)	0.4 (0.3-0.5)	3	0.03	0.06 (0.01-0.1)	0.1 (0.02-0.1)
	Fatal	4	0.04	0.1 (0.02-0.1)	0.1 (0.04-0.2)	6	0.06	0.1 (0.1-0.2)	0.1 (0.1-0.2)	2	0.02	0.04 (0-0.08)	0.05 (0-0.1)
All	ICH	21	0.2	0.4 (0.2-0.5)	0.4 (0.3-0.5)	16	0.2	0.3 (0.2-0.4)	0.3 (0.2-0.5)	3	0.03	0.05 (0-0.1)	0.06 (0.01-0.1)
	Fatal	4	0.04	0.1 (0.02-0.1)	0.1 (0.02-0.1)	6	0.06	0.1 (0.04 - 0.2)	0.1 (0.05-0.2)	2	0.02	0.03 (0-0.07)	0.04 (0-0.1)

All DOACS (n=8972)

Time window	Outcome	Intracerebral/intraventricular hemorrhage				Subarachnoid hemorrhage				Subdural, epidural and extradural hemorrhages			
		Outcomes (n)	Outcomes (%)	Event rate (95% C)	IR (95% CI)	Outcomes (n)	Outcomes (%)	Event rate (95% C)	IR (95% CI)	Outcomes (n)	Outcomes (%)	Event rate (95% C)	IR (95% CI)
3 months	ICH	7	0.08	0.4 0.3-0.6)	0.4 (0.3-0.6)	8	0.1	0.5 (0.4-0.6)	0.5 (0.4-0.7)	2	0.02	0.1 (0.05-0.2)	0.1 (0.05-0.2)
	Fatal	2	0.02	0.1 (0.05-0.2)	0.1 (0.05-0.2)	2	0.02	0.1 (0.05-0.2)	0.1 (0.05-0.2)	2	0.02	0.1 (0.05-0.2)	0.1 (0.05-0.2)
6 months	ICH	11	0.1	0.5 (0.3-0.6)	0.5 (0.3-0.6)	10	0.1	0.4 (0.3-0.6)	0.4 (0.3-0.6)	3	0.03	0.1 (0.06-0.2)	0.1 (0.06-0.2)
	Fatal	2	0.02	0.1 (0.02-0.1)	0.1 (0.03-0.2)	3	0.03	0.1 (0.06-0.2)	0.1 (0.06-0.2)	2	0.02	0.1 (0.02-0.1)	0.1 (0.03-0.2)
12 months	ICH	14	0.2	0.5 (0.3-0.6)	0.5 (0.3-0.6)	10	0.1	0.3 (0.2-0.5)	0.4 (0.2-0.5)	3	0.03	0.1 (0.03-0.2)	0.1 (0.03-0.2)
	Fatal	2	0.02	0.1 (0.02-0.1)	0.1 (0.02-0.1)	3	0.03	0.1 (0.03-0.2)	0.1 (0.03-0.2)	2	0.02	0.1 (0.02-0.1)	0.07 (0.02-0.1)
2 years	ICH	14	0.2	0.4 (0.2-0.5)	0.4 (0.2-0.5)	13	0.1	0.3 (0.2-0.5)	0.4 (0.2-0.5)	3	0.03	0.1 (0.02-0.1)	0.1 (0.02-0.1)
	Fatal	2	0.02	0.05 (0-0.1)	0.06 (0.01-0.1)	4	0.04	0.1 (0.03-0.2)	0.1 (0.04-0.2)	2	0.02	0.05 (0-0.1)	0.06 (0.01-0.1)
3 years	ICH	16	0.2	0.4 (0.2-0.5)	0.4 (0.2-0.5)	13	0.1	0.3 (0.2-0.4)	0.3 (0.2-0.4)	3	0.03	0.1 (0.02-0.1)	0.07 (0.02-0.1)
	Fatal	3	0.03	0.1 (0.02-0.1)	0.1 (0.02-0.1)	4	0.04	0.1 (0.03-0.2)	0.1 (0.03-0.2)	2	0.02	0.04 (0-0.1)	0.05 (0-0.1)
All	ICH	17	0.2	0.3 (0.2-0.4)	0.3 (0.2-0.5)	13	0.1	0.2 (0.1-0.3)	0.3 (0.2-0.4)	3	0.03	0.05 (0-0.1)	0.06 (0.01-0.1)
	Fatal	3	0.03	0.05 (0-0.1)	0.06 (0.01-0.1)	4	0.04	0.07 (0.02-0.1)	0.1 (0.03-0.2)	2	0.02	0.03 (0-0.1)	0.04 (0-0.1)

Enoxaparin (n=402)

Time window	Outcome	Intracerebral/intraventricular hemorrhage				Subarachnoid hemorrhage				Subdural, epidural and extradural hemorrhages			
		Outcomes (n)	Outcomes (%)	Event rate (95% C)	IR (95% CI)	Outcomes (n)	Outcomes (%)	Event rate (95% C)	IR (95% CI)	Outcomes (n)	Outcomes (%)	Event rate (95% C)	IR (95% CI)
3 months	ICH	3	0.8	4.3 (2.4-6.3)	4.4 (2.4-6.4)	0	0	0	0	0	0	0	0
	Fatal	1	0.3	1.5 (0.3-2.6)	1.5 (0.3-2.7)	0	0	0	0	0	0	0	0
6 months	ICH	3	0.8	3.2 (1.5-4.9)	3.3 (1.56-5.1)	1	0.3	1.1 (0.06-2.1)	1.1 (0.1-2.1)	0	0	0	0
	Fatal	1	0.3	1.1 (0.06-2.1)	1.1 (0.1-2.1)	1	0.3	1.1 (0.06-2.1)	1.1 (0.1-2.1)	0	0	0	0
12 months	ICH	3	0.8	2.6 (1.0-4.1)	2.7 (1.1-4.3)	3	0.8	2.6 (1.0-4.1)	2. (1.1-4.3)	0	0	0	0
	Fatal	1	0.3	0.9 (0-1.8)	0.9 (0-1.8)	2	0.5	1.7 (0.4-3.0)	1.8 (0.5-3.1)	0	0	0	0
2 years	ICH	3	0.8	1.9 (0.6-3.3)	2.1 (0.7-3.5)	3	0.8	1.9 (0.6-3.3)	2.1 (0.7-3.5)	0	0	0	0
	Fatal	1	0.3	0.7 (0-1.4)	0.7 (0-1.5)	2	0.5	1.3 (0.2-2.4)	1.4 (0.2-2.5)	0	0	0	0
3 years	ICH	4	1.0	2.2 (0.8-3.7)	2.5 (1.0 -4.0)	3	0.8	1.7 (0.4-2.9)	1.9 (0.5-3.2)	0	0	0	0
	Fatal	1	0.3	0.6 (0-1.3)	0.6 (0-1.4)	2	0.5	1.1 (0.1-2.1)	1.2 (0.2-2.3)	0	0	0	0
All	ICH	4	1.0	1.6 (0.4-2.9)	2.0 (0.6-3.4)	3	0.8	1.2 (0.2-2.3)	1.5 (0.3-2.7)	0	0	0	0
	Fatal	1	0.3	0.4 (0-1.0)	0.5 (0-1.2)	2	0.5	0.8 (0-1.7)	1.0 (0.03-2.0)	0	0	0	0

DOACs: direct oral anticoagulants; FXai; Factor Xa inhibitors; ICH: intracranial hemorrhage; IR (95% CI): Incidence rate (95% confidence interval). Time window: Cumulative bleedings from index (day of the first recording with DOAC or enoxaparin in active oncology patients) to the specific time window; Outcome: first bleeding; Event rate (events per 100 person-years) was defined as as the total number of bleeding events, including recurrent events divided by the total person-time of follow-up. Incidence rate (IR, events per 100 person-years) was defined as the total number of incident bleeding events divided by the total person-time at risk; Enoxaparin group: active oncology patients only; N: number of patients in the total group or subgroup or with the event.

Supplementary Table S8. Trauma-related bleeding event and incidence rates.

All FXai (n=9374)

Time window	Outcome	Overall TRMB				Trauma intracranial hemorrhage				Other TRMB			
		Outcomes (n)	Outcomes (%)	Event rate (95% C)	IR (95% CI)	Outcomes (n)	Outcomes (%)	Event rate (95% C)	IR (95% CI)	Outcomes (n)	Outcomes (%)	Event rate (95% C)	IR (95% CI)
3 months	TRMB	6	0.06	0.4 (0.2-0.5)	0.4 (0.2-0.5)	4	0.04	0.2 (0.1-0.3)	0.2 (0.1-0.3)	2	0.02	0.1 (0.05-0.2)	0.1 (0.05-0.2)
	Fatal	5	0.05	0.3 (0.2-0.4)	0.3 (0.19 - 0.41)	4	0.04	0.2 (0.1-0.3)	0.2 (0.1-0.3)	1	0.01	0.06 (0.01-0.1)	0.06 (0.01-0.1)
6 months	TRMB	9	0.1	0.4 (0.3-0.5)	0.38 (0.26 - 0.5)	6	0.06	0.3 (0.2-0.4)	0.3 (0.2-0.4)	3	0.03	0.1 (0.05-0.2)	0.1 (0.06-0.2)
	Fatal	7	0.07	0.3 (0.2-0.4)	0.29 (0.18 - 0.4)	5	0.05	0.2 (0.1-0.3)	0.2 (0.1-0.3)	2	0.02	0.1 (0.02-0.1)	0.08 (0.02-0.1)
12 months	TRMB	9	0.1	0.3 (0.2-0.4)	0.3 (0.19 - 0.41)	6	0.06	0.2 (0.1-0.3)	0.2 (0.1-0.3)	3	0.03	0.1 (0.04-0.2)	0.1 (0.04-0.2)
	Fatal	7	0.07	0.2 (0.1-0.3)	0.23 (0.13 - 0.33)	5	0.05	0.2 (0.1-0.2)	0.2 (0.1-0.3)	2	0.02	0.06 (0.01-0.1)	0.07 (0.02-0.1)
2 years	TRMB	10	0.1	0.3 (0.2-0.4)	0.27 (0.16 - 0.38)	7	0.07	0.2 (0.1-0.3)	0.2 (0.1-0.3)	3	0.03	0.07 (0.02-0.1)	0.08 (0.02-0.1)
	Fatal	8	0.1	0.2 (0.1-0.3)	0.21 (0.12 - 0.3)	6	0.06	0.2 (0.1-0.2)	0.2 (0.1-0.2)	2	0.02	0.05 (0-0.1)	0.05 (0-0.1)
3 years	TRMB	11	0.1	0.2 (0.1-0.3)	0.26 (0.16 - 0.36)	7	0.07	0.2 (0.1-0.2)	0.2 (0.1-0.3)	4	0.04	0.1 (0.02-0.1)	0.1 (0.04-0.2)
	Fatal	8	0.1	0.2 (0.1-0.3)	0.19 (0.1 - 0.28)	6	0.06	0.1 (0.06-0.2)	0.1 (0.06-0.2)	2	0.02	0.04 (0-0.08)	0.05 (0-0.1)
All	TRMB	12	0.1	0.2 0.1-0.3)	0.23 (0.13 - 0.33)	7	0.07	0.1 (0.05-0.2)	0.1 (0.06-0.2)	5	0.05	0.1 (0.02-0.1)	0.08 (0.02-0.1)
	Fatal	8	0.1	0.1 (0.06-0.2)	0.16 (0.08 - 0.24)	6	0.06	0.1 (0.04-0.2)	0.1 (0.05-0.2)	2	0.02	0.03 (0-0.07)	0.04 (0-0.08)

All DOACS (n=8972)

Time window	Outcome	Overall TRMB				Trauma intracranial hemorrhage				Other TRMB			
		Outcomes (n)	Outcomes (%)	Event rate (95% C)	IR (95% CI)	Outcomes (n)	Outcomes (%)	Event rate (95% C)	IR (95% CI)	Outcomes (n)	Outcomes (%)	Event rate (95% C)	IR (95% CI)
3 months	TRMB	5	0.06	0.3 (0.2-0.4)	0.3 (0.2-0.4)	3	0.03	0.2 (0.1-0.3)	0.2 (0.1-0.3)	2	0.02	0.1 (0.05-0.2)	0.1 (0.05-0.2)
	Fatal	4	0.04	0.2 (0.1-0.3)	0.3 (0.2-0.4)	3	0.03	0.2 (0.1-0.3)	0.2 (0.1-0.3)	1	0.01	0.06 (0.01-0.1)	0.06 (0.01-0.1)
6 months	TRMB	8	0.1	0.3 (0.2-0.5)	0.4 (0.2-0.5)	5	0.06	0.2 (0.1-0.3)	0.2 (0.1-0.3)	3	0.03	0.1 (0.06-0.2)	0.1 (0.06-0.2)
	Fatal	6	0.07	0.3 (0.2-0.4)	0.3 (0.2-0.4)	4	0.04	0.2 (0.1-0.3)	0.2 (0.1-0.3)	2	0.02	0.08 (0.02-0.1)	0.1 (0.03-0.2)
12 months	TRMB	8	0.1	0.3 (0.2-0.4)	0.3 (0.2-0.4)	5	0.06	0.2 (0.1-0.2)	0.2 (0.1-0.3)	3	0.03	0.1 (0.03-0.2)	0.1 (0.03-0.2)
	Fatal	6	0.07	0.2 (0.1-0.3)	0.2 (0.1-0.3)	4	0.04	0.1 (0.06-0.2)	0.1 (0.06-0.2)	2	0.02	0.07 (0.02-0.1)	0.07 (0.02-0.1)
2 years	TRMB	9	0.1	0.2 (0.1-0.3)	0.3 (0.2-0.4)	6	0.07	0.2 (0.07-0.2)	0.2 (0.08-0.3)	3	0.03	0.08 (0.02-0.1)	0.08 (0.02-0.1)
	Fatal	7	0.1	0.2 (0.1-0.3)	0.2 (0.1-0.3)	5	0.06	0.1 (0.06-0.2)	0.1 (0.06-0.2)	2	0.02	0.05 (0-0.1)	0.06 (0.01-0.1)
3 years	TRMB	10	0.1	0.2 (0.1-0.3)	0.3 (0.2-0.4)	6	0.07	0.1 (0.06-0.2)	0.2 (0.07-0.2)	4	0.04	0.1 (0.03-0.2)	0.1 (0.03-0.2)
	Fatal	7	0.01	0.2 (0.1-0.2)	0.2 (0.1-0.3)	5	0.06	0.1 (0.04-0.2)	0.1 (0.05-0.2)	2	0.02	0.04 (0-0.08)	0.05 (0-0.1)
All	TRMB	11	0.1	0.2 (0.1-0.3)	0.2 (0.1-0.3)	6	0.07	0.1 (0.03-0.2)	0.1 (0.06-0.2)	5	0.06	0.1 (0.03-0.2)	0.09 (0.03-0.2)
	Fatal	7	0.1	0.1 (0.05-0.2)	0.2 (0.1-0.2)	5	0.06	0.1 (0.03-0.2)	0.1 (0.04-0.2)	2	0.02	0.03 (0-0.07)	0.04 (0-0.08)

Enoxaparin (n=402)

Time window	Outcome	Overall TRMB				Trauma intracranial hemorrhage				Other TRMB			
		Outcomes (n)	Outcomes (%)	Event rate (95% C)	IR (95% CI)	Outcomes (n)	Outcomes (%)	Event rate (95% C)	IR (95% CI)	Outcomes (n)	Outcomes (%)	Event rate (95% C)	IR (95% CI)
3 months	TRMB	1	0.3	1.5 (0.3-2.6)	1.5 (0.3-2.7)	1	0.3	1.5 (0.3-2.6)	1.5 (0.3-2.7)	0	0	0	0
	Fatal	1	0.3	1.5 (0.3-2.6)	1.5 (0.3-2.7)	1	0.3	1.5 (0.3-2.6)	1.5 (0.3-2.7)	0	0	0	0
6 months	TRMB	1	0.3	1.1 (0.06-2.1)	1.1 (0.08-2.1)	1	0.3	1.1 (0.06-2.1)	1.1 (0.08-2.1)	0	0	0	0
	Fatal	1	0.3	1.1 (0.06-2.1)	1.1 (0.08-2.1)	1	0.3	1.1 (0.06-2.1)	1.1 (0.08-2.1)	0	0	0	0
12 months	TRMB	1	0.3	0.9 (0-1.8)	0.9 (0-1.8)	1	0.3	0.8 (0-1.8)	0.9 (0-1.8)	0	0	0	0
	Fatal	1	0.3	0.9 (0-1.8)	0.9 (0-1.8)	1	0.3	0.9 (0-1.8)	0.9 (0-1.8)	0	0	0	0
2 years	TRMB	1	0.3	0.7 (0-1.4)	0.7 (0-1.5)	1	0.3	0.7 (0-1.4)	0.7 (0-1.5)	0	0	0	0
	Fatal	1	0.3	0.7 (0-1.4)	0.7 (0-1.5)	1	0.3	0.7 (0-1.4)	0.7 (0-1.5)	0	0	0	0
3 years	TRMB	1	0.3	0.6 (0-1.3)	0.6 (0-1.4)	1	0.3	0.6 (0-1.3)	0.6 (0-1.4)	0	0	0	0
	Fatal	1	0.3	0.6 (0-1.3)	0.6 (0-1.4)	1	0.3	0.6 (0-1.3)	0.6 (0-1.4)	0	0	0	0
All	TRMB	1	0.3	0.4 (0-1.0)	0.5 (0-1.2)	1	0.3	0.4 (0-1.0)	0.5 (0-1.2)	0	0	0	0
	Fatal	1	0.3	0.4 (0-1.0)	0.5 (0-1.2)	1	0.3	0.4 (0-1.0)	0.5 (0-1.2)	0	0	0	0

DOACs: direct oral anticoagulants; FXai; Factor Xa inhibitors; TRMB: Trauma related major bleeding; IR (95% CI): Incidence rate (95% confidence interval). Time window: Cumulative bleedings from index (day of the first recording with DOAC or enoxaparin in active oncology patients) to the specific time window; Outcome: first bleeding; Event rate (events per 100 person-years) was defined as as the total number of bleeding events, including recurrent events divided by the total person-time of follow-up. Incidence rate (IR, events per 100 person-years) was defined as the total number of incident bleeding events divided by the total person-time at risk; Enoxaparin group: active oncology patients only; N: number of patients in the total group or subgroup or with the event.

Supplementary Table S9. Other major bleeding event and incidence rates.

All FXai (n=9374)

Time window	Outcome	Other MB				Genitourinary MB				Respiratory tract MB				Unspecified other MB			
		Outcomes (n)	Outcomes (%)	Event rate (95% C)	IR (95% CI)	Outcomes (n)	Outcomes (%)	Event rate (95% C)	IR (95% CI)	Outcom es (n)	Outcomes (%)	Event rate (95% C)	IR (95% CI)	Outcomes (n)	Outcomes (%)	Event rate (95% C)	IR (95% CI)
3 months	Other MB	15	0.2	0.9 (0.7-1.1)	0.9 (0.7-1.1)	9	0.10	0.5 (0.4-0.7)	0.5 (0.4-0.7)	4	0.0%	0.2 (0.1-0.3)	0.24 (0.1-0.3)	2	0.02	0.1 (0.05-0.2)	0.1 (0.05-0.2)
	Fatal	1	0.01	0.06 (0.01-0.1)	0.06 (0.01-0.1)	0	0	0	0	0	0	0	0	1	0.01	0.06 (0.01-0.1)	0.06 (0.01-0.1)
6 months	Other MB	19	0.2	0.8 (0.6-1.0)	0.8 (0.6-1.0)	11	0.1	0.5 (0.3-0.6)	0.5 (0.3-0.6)	4	0.04	0.2 (0.08-0.2)	0.2 (0.09-0.3)	4	0.04	0.2 (0.08-0.2)	0.2 (0.09-0.3)
	Fatal	2	0.02	0.08 (0.02-0.1)	0.08 (0.02-0.1)	1	0.01	0.04 (0-0.08)	0.04 (0-0.08)	0	0	0	0	1	0.01	0.04 (0-0.08)	0.04 (0-0.08)
12 months	Other MB	19	0.2	0.6 (0.4-0.8)	0.6 (0.5-0.8)	11	0.12	0.4 (0.2-0.5)	0.4 (0.3-0.5)	4	0.04	0.1 (0.06-0.2)	0.1 (0.06-0.2)	4	0.04	0.1 (0.06-0.2)	0.1 (0.06-0.2)
	Fatal	2	0.02	0.06 (0.01-0.1)	0.07 (0.02-0.1)	1	0.01	0.03 (0-0.07)	0.03 (0-0.07)	0	0	0	0	1	0.01	0.03 (0-0.07)	0.03 (0-0.07)
2 years	Other MB	20	0.2	0.5 (0.4-0.6)	0.5 (0.4 0.7)	12	0.1	0.3 (0.2-0.4)	0.3 (0.2-0.4)	4	0.04	0.1 (0.04-0.2)	0.1 (0.04-0.2)	4	0.04	0.1 (0.04-0.2)	0.1 (0.04-0.2)
	Fatal	2	0.02	0.05 (0-0.1)	0.05 (0-0.1)	1	0.01	0.02 (0-0.05)	0.03 (0-0.07)	0	0	0	0	1	0.01	0.02 (0-0.05)	0.03 (0-0.07)
3 years	Other MB	22	0.2	0.5 (0.3-0.6)	0.5 (0.4-0.6)	13	0.1	0.3 (0.2-0.4)	0.3 (0.2-0.4)	4	0.04	0.08 (0.02-0.1)	0.1 (0.04-0.2)	4	0.04	0.08 (0.02-0.1)	0.1 (0.04-0.2)
	Fatal	2	0.02	0.04 (0-0.08)	0.05 (0-0.1)	1	0.01	0.02 (0-0.05)	0.02 (0-0.05)	0	0	0	0	1	0.01	0.02 (0-0.05)	0.02 (0-0.05)
All	Other MB	23	0.3	0.4 (0.3-0.5)	0.4 (0.3-0.6)	13	0.1	0.2 (0.1-0.3)	0.3 (0.2-0.4)	4	0.04	0.07 (0.02-0.1)	0.08 (0.02-0.1)	5	0.05	0.08 (0.02-0.1)	0.08 (0.02-0.1)
	Fatal	2	0.02	0.03 (0-0.07)	0.04 (0-0.08)	1	0.01	0.02 (0-0.05)	0.02 (0-0.05)	0	0	0	0	1	0.01	0.02 (0-0.05)	0.02 (0-0.05)

All DOACS (n=8972)

Time window	Outcome	Other MB				Genitourinary MB				Respiratory tract MB				Unspecified other MB			
		Outcomes (n)	Outcomes (%)	Event rate (95% C)	IR (95% CI)	Outcomes (n)	Outcomes (%)	Event rate (95% C)	IR (95% CI)	Outcom es (n)	Outcomes (%)	Event rate (95% C)	IR (95% CI)	Outcomes (n)	Outcomes (%)	Event rate (95% C)	IR (95% CI)
3 months	Other MB	15	0.2	0.9 (0.7-1.1)	0.9 (0.7-1.1)	9	0.1	0.6 (0.4-0.7)	0.6 (0.4-0.7)	4	0.04	0.2 (0.1-0.3)	0.3 (0.2-0.4)	2	0.02	0.2 (0.05-0.2)	0.1 (0.05-0.2)
	Fatal	1	0.01	0.06 (0.01-0.1)	0.06 (0.01-0.1)	0	0	0	0	0	0	0	0	1	0.01	0.06 (0.01-0.1)	0.06 (0.01-0.1)
6 months	Other MB	19	0.2	0.8 (0.6-1)	0.8 (0.6-1.0)	11	0.1	0.5 (0.3-0.6)	0.5 (0.3-0.6)	4	0.04	0.2 (0.08-0.3)	0.2 (0.1-0.3)	4	0.04	0.2 (0.1-0.3)	0.2 (0.08-0.3)
	Fatal	2	0.02	0.08 (0.02-0.1)	0.1 (0.03-0.2)	1	0.01	0.04 (0-0.08)	0.04 (0-0.08)	0	0	0	0	1	0.01	0.04 (0-0.08)	0.04 (0-0.08)
12 months	Other MB	19	0.2	0.6 (0.5-0.8)	0.7 (0.5-0.8)	11	0.1	0.4 (0.2-0.5)	0.4 (0.3-0.5)	4	0.04	0.1 (0.06-0.2)	0.1 (0.06-0.2)	4	0.04	0.1 (0.06-0.2)	0.1 (0.06-0.2)
	Fatal	2	0.02	0.07 (0.02-0.1)	0.07 (0.02-0.1)	1	0.01	0.03 (0-0.07)	0.03 (0-0.07)	0	0	0	0	1	0.01	0.03 (0-0.07)	0.03 (0-0.07)
2 years	Other MB	19	0.2	0.5 (0.3-0.6)	0.5 (0.4-0.7)	11	0.1	0.3 (0.2-0.4)	0.3 (0.2-0.4)	4	0.04	0.1 (0.03-0.2)	0.1 (0.04-0.2)	4	0.04	0.1 (0.03-0.2)	0.1 (0.04-0.2)
	Fatal	2	0.02	0.05 (0-0.1)	0.06 (0.01-0.1)	1	0.01	0.03 (0-0.07)	0.03 (0-0.07)	0	0	0	0	1	0.01	0.03 (0-0.07)	0.03 (0-0.07)
3 years	Other MB	21	0.2	0.5 (0.3-0.6)	0.5 (0.4-0.7)	12	0.1	0.3 (0.2-0.4)	0.3 (0.2-0.4)	4	0.04	0.09 (0.03-0.2)	0.1 (0.03-0.2)	4	0.04	0.09 (0.03-0.2)	0.1 (0.03-0.2)
	Fatal	2	0.02	0.04 (0-0.08)	0.05 (0-0.1)	1	0.01	0.02 (0-0.05)	0.02 (0-0.05)	0	0	0	0	1	0.01	0.02 (0-0.05)	0.02 (0-0.05)
All	Other MB	22	0.3	0.4 (0.3-0.5)	0.4 (0.3-0.6)	12	0.1	0.2 (0.1-0.3)	0.3 (0.2-0.4)	4	0.04	0.07 (0.02-0.1)	0.1 (0.03-0.2)	5	0.06	0.1 (0.03-0.2)	0.1 (0.03-0.2)
	Fatal	2	0.02	0.03 (0-0.07)	0.04 (0-0.08)	1	0.01	0.02 (0-0.05)	0.02 (0-0.05)	0	0	0	0	1	0.01	0.02 (0-0.05)	0.02 (0-0.05)

Enoxaparin (n=402)

Time window	Outcome	Other MB				Genitourinary MB				Respiratory tract MB				Unspecified other MB			
		Outcomes (n)	Outcomes (%)	Event rate (95% C)	IR (95% CI)	Outcomes (n)	Outcomes (%)	Event rate (95% C)	IR (95% CI)	Outcom es (n)	Outcomes (%)	Event rate (95% C)	IR (95% CI)	Outcomes (n)	Outcomes (%)	Event rate (95% C)	IR (95% CI)
3 months	Other MB	0	0	0	0	0	0	0	0	0	0	0	0	0	0	0	0
	Fatal	0	0	0	0	0	0	0	0	0	0	0	0	0	0	0	0
6 months	Other MB	0	0	0	0	0	0	0	0	0	0	0	0	0	0	0	0
	Fatal	0	0	0	0	0	0	0	0	0	0	0	0	0	0	0	0
12 months	Other MB	0	0	0	0	0	0	0	0	0	0	0	0	0	0	0	0
	Fatal	0	0	0	0	0	0	0	0	0	0	0	0	0	0	0	0
2 years	Other MB	1	0.3	0.7 (0-1.4)	0.7 (0-1.5)	1	0.3	0.7 (0-1.4)	0.7 (0-1.5)	0	0	0	0	0	0	0	0
	Fatal	0	0	0	0	0	0	0	0	0	0	0	0	0	0	0	0
3 years	Other MB	1	0.3	0.6 (0-1.3)	0.6 (0-1.4)	1	0.3	0.6 (0-1.3)	0.6 (0-1.4)	0	0	0	0	0	0	0	0
	Fatal	0	0	0	0	0	0	0	0	0	0	0	0	0	0	0	0
All	Other MB	1	0.3	0.4 (0-1.0)	0.5 (0-1.2)	1	0.3	0.4 (0-1.0)	0.5 (0-1.2)	0	0	0	0	0	0	0	0
	Fatal	0	0	0	0	0	0	0	0	0	0	0	0	0	0	0	0

DOACs: direct oral anticoagulants; FXai; Factor Xa inhibitors; MB: major bleeding; IR (95% CI): Incidence rate (95% confidence interval). Time window: Cumulative bleedings from index (day of the first recording with DOAC or enoxaparin in active oncology patients) to the specific time window; Outcome: first bleeding; Event rate (events per 100 person-years) was defined as as the total number of bleeding events, including recurrent events divided by the total person-time of follow-up. Incidence rate (IR, events per 100 person-years) was defined as the total number of incident bleeding events divided by the total person-time at risk; Enoxaparin group: active oncology patients only; N: number of patients in the total group or subgroup or with the event.

Supplementary Table S10. Risk factors for major bleeds using COX regression models for DOAC patients.

	OR	95% CI	P
**Biodemographic data**			
Age at index date (years)	1.06	1.05-1.07	<0.01
Gender (male vs female)	0.78	0.64-0.94	0.01
BMI	0.99	0.97-1.01	0.35
Alcohol use	1.12	0.75-1.67	0.58
Updated Charlson index	0.80	0.64-1.00	0.05
**Cardiovascular risk factors**			
Hypertension	1.03	0.75-1.41	0.85
Hypercholesterolemia	1.07	0.86-1.33	0.55
Diabetes type 2	1.30	0.66-2.56	0.44
Diabetes type 1	1.75	0.68-4.50	0.25
Smoking	1.14	0.83-1.55	0.42
**Vascular disease**			
Heart failure	0.94	0.55-1.58	0.81
Chronic kidney disease	0.69	0.24-1.99	0.49
Coronary artery disease	0.80	0.52-1.22	0.29
Myocardial infarction	1.43	0.85-2.42	0.18
Peripheral vascular disease	1.43	0.96-2.13	0.08
Cerebrovascular disease	1.41	0.17-11.88	0.75
**Other comorbidities**			
Chronic pulmonary disease	1.18	0.86-1.62	0.31
Cancer	1.49	0.69-3.21	0.32
Anemia	1.13	0.77-1.64	0.54
Major bleeding history >60 days prior to FXai start	1.21	0.90-1.63	0.21
Moderate or severe liver disease	1.33	0.36-4.90	0.67
Peptic ulcer disease	1.53	0.93-2.54	0.10
**FXai use**			
Calendar year of first FXai use			
2014	1.75	0.68-4.52	0.244
2015	1.51	0.60-3.83	0.384
2016	0.89	0.33-2.39	0.825
2017	1.82	0.76-4.38	0.178
2018	2.05	0.88-4.80	0.097
2019	1.85	0.79-4.34	0.157
2020	1.84	0.79-4.27	0.158
2021	1.75	0.76-4.01	0.187
2022	2.29	1.00-5.23	0.050
FXai indications			
AF (reference)	-	-	-
VTE	0.76	0.60-0.97	0.03
Non-mechanical cardiac-valve replacement	0.78	0.49-1.23	0.29
DOAC dose as per label			
No dose reduction	0.85	0.66-1.09	0.20
Dose reduction as per label	0.88	0.67-1.16	0.37
Medical possesion ratio to FXai (% prescribed doses taken)	0.99	0.96-1.01	0.32
Compliance ≥80%	1.14	0.74-1.77	0.56
Discontinuation after MB	5.33	3.99-7.12	<0.01
**Concomitant treatments (within 120 days prior to index)**			
Gastroprotective agents	0.94	0.77-1.15	0.55
Antihypertensive therapies	1.08	0.83-1.41	0.56
Lipid lowering drugs	1.08	0.85-1.37	0.54
Antidiabetic drugs	1.42	0.65-3.10	0.39
NSAIDs	1.16	0.95-1.41	0.14
Antiplatelet drugs	0.89	0.69-1.16	0.39
Anticancer drugs*	1.26	0.62-2.54	0.52
**Biochemical parameters**			
Hemoglobin	0.96	0.94-0.99	0.01
HbA1c	0.96	0.89-1.04	0.34
Platelet count	1.00	1.00-1.00	0.79
eGFR	1.00	0.99-1.01	0.99
Creatinine clearance	1.00	0.99-1.00	0.03

*within 180 days prior index. AF: atrial fibrillation; BMI: body mass index; DOACs: direct oral anticoagulants; eGFR: estimated glomerular filtration rate; FXai; Factor Xa inhibitors; NSAIDs: non-steroidal anti-inflammatory drugs; OR: Odds Ratio; 95% CI: 95% confidence interval; VTE: Venous thromboembolism.
